# Shared genetic architecture of schizophrenia and lupus identifies pleiotropic loci with distinct downstream mechanisms

**DOI:** 10.1093/hmg/ddag099

**Published:** 2026-10-01

**Authors:** Chen Gong, Nick Dand, Amy W Butler, Ariella Amar, Lu Liu, Pinelopi Maria Triantafyllou, David L Morris, Timothy J Vyse

**Affiliations:** Department of Medical & Molecular Genetics, King’s College London, St Thomas Street, London, SE1 9RT, United Kingdom; Department of Medical & Molecular Genetics, King’s College London, St Thomas Street, London, SE1 9RT, United Kingdom; Department of Medical & Molecular Genetics, King’s College London, St Thomas Street, London, SE1 9RT, United Kingdom; Department of Medical & Molecular Genetics, King’s College London, St Thomas Street, London, SE1 9RT, United Kingdom; Department of Dermatology, The First Affiliated Hospital of Anhui Medical University, Hefei, Anhui, China; Department of Medical & Molecular Genetics, King’s College London, St Thomas Street, London, SE1 9RT, United Kingdom; Department of Medical & Molecular Genetics, King’s College London, St Thomas Street, London, SE1 9RT, United Kingdom; Department of Medical & Molecular Genetics, King’s College London, St Thomas Street, London, SE1 9RT, United Kingdom

**Keywords:** Schizophrenia, systemic lupus erythematosus, pleiotropy, genetic correlation, GWAS, eQTL, FURIN, FES

## Abstract

Schizophrenia (SCZ) and systemic lupus erythematosus (SLE) exhibit epidemiological overlap, suggesting shared genetic underpinnings. Using the largest SLE genome-wide association study (GWAS) to date, we identified a significant genetic correlation between SCZ and SLE (r_g_ = 0.25, p = 9.0 × 10^−4^). Cross-trait analysis nominated multiple candidate shared loci outside the major histocompatibility complex (MHC), most with aligned effects. The strongest candidate shared association was found at the *FURIN-FES* locus, previously implicated in SCZ and newly associated with SLE. In addition to *FURIN-FES*, *FCGR2A*, *DOT1L* and an intergenic noncoding RNA locus on chromosome 2 showed additional evidence consistent with shared association. Functional annotation showed that this locus modulated the expression of *FURIN*, which encodes a proprotein convertase involved in diverse biological processes, and *FES*, which encodes a tyrosine kinase important in innate immune signalling. The shared risk allele was also associated with altered plasma levels of FURIN, FES, and their functional partners (GDF2, BMP10, NTF4, FER). Tissue- and cell-type-specific eQTL analysis revealed distinct regulatory effects: *FURIN* expression was perturbed in neuronal and immune cells, while *FES* expression was altered in immune cells. Mendelian randomisation supported a causal role for FES protein in both disorders. These findings highlight that pleiotropy at the *FURIN-FES* locus for SCZ and SLE is compatible with disease-specific mechanisms. This underscores that shared genetics across traits does not necessarily mean shared biology.

## Introduction

Schizophrenia (SCZ) is a severe psychiatric disorder characterised by chronic psychosis, cognitive impairments, and other neuropsychiatric symptoms. Although traditionally viewed as a disorder rooted in neurodevelopmental abnormalities, recent research [1] has highlighted a potential link between SCZ and immune dysfunction. Systemic Lupus Erythematosus (SLE), a prototypic autoimmune disease that affects multiple organ systems, including the central nervous system, has been observed to co-occur with SCZ at higher rates than would be expected by chance [2, 3]. This epidemiological overlap raises the possibility of shared genetic and pathophysiological mechanisms between SCZ and SLE.

Genetic overlap between SLE and SCZ has been observed at the individual gene level with copy number variation at complement *C4* [4]. In this case the relationship is reciprocal, with elevated *C4* copy number increasing the risk of SCZ, whilst reducing the risk of SLE. Genome-wide association studies (GWAS) have identified numerous risk loci for both SCZ and autoimmune diseases. A recent comprehensive analysis of SCZ and 19 immune-mediated diseases (IMD), including SLE, identified significant genetic correlations between SCZ and several IMD [5]. For instance, genetic overlap was observed between SCZ and SLE (rg = 0.13, p = 3.76 × 10^−3^), as well as with other diseases such as Crohn’s disease (CRO), inflammatory bowel disease (IBD), and psoriasis (PSO). This study highlighted the potential role of inflammation and immune dysregulation in the pathogenesis of SCZ.

We build on these findings by investigating the shared genetics of SCZ and SLE using large-scale GWAS data [6–10]. We aim to systematically evaluate the extent and nature of their shared genetic architecture, then identify specific loci contributing to this overlap, and investigate the underlying biological mechanisms. To achieve this, we combined large-scale GWAS datasets for both disorders to perform genome-wide analyses of genetic correlation and cross-trait meta-analysis. We then performed regional and functional follow-up analyses to characterise candidate shared loci, incorporating higher-resolution data from Immunochip alongside colocalisation and functional annotation using eQTL and pQTL resources. This approach allowed us to identify candidate shared loci and assess whether observed genetic overlap is consistent with shared causal variants and convergent biological mechanisms.

## Results

### Genetic correlation and cross-trait enrichment

We investigated the genome-wide genetic correlation between SCZ and SLE through LD Score Regression (LDSC) [11] using the ancestry-matched SLE and SCZ summary association statistics, with the LD scores estimated from 1000 Genomes European samples and excluding the extended major histocompatibility complex (MHC) region (see Materials and Methods for details). We observed a significant positive genetic correlation (r_g_ = 0.25, s.e. = 0.074, p = 9.0 × 10^−4^).

Q-Q plots of SNPs published as associated with one trait tested in the other (see methods) showed modest deviation from the null, consistent with enrichment of cross-disease association signals (Fig. 1). A small number of outliers were observed, most notably at the *FURIN–FES* locus, which showed the strongest evidence of cross-trait association. (Supplementary Table 1).

**Figure 1:**
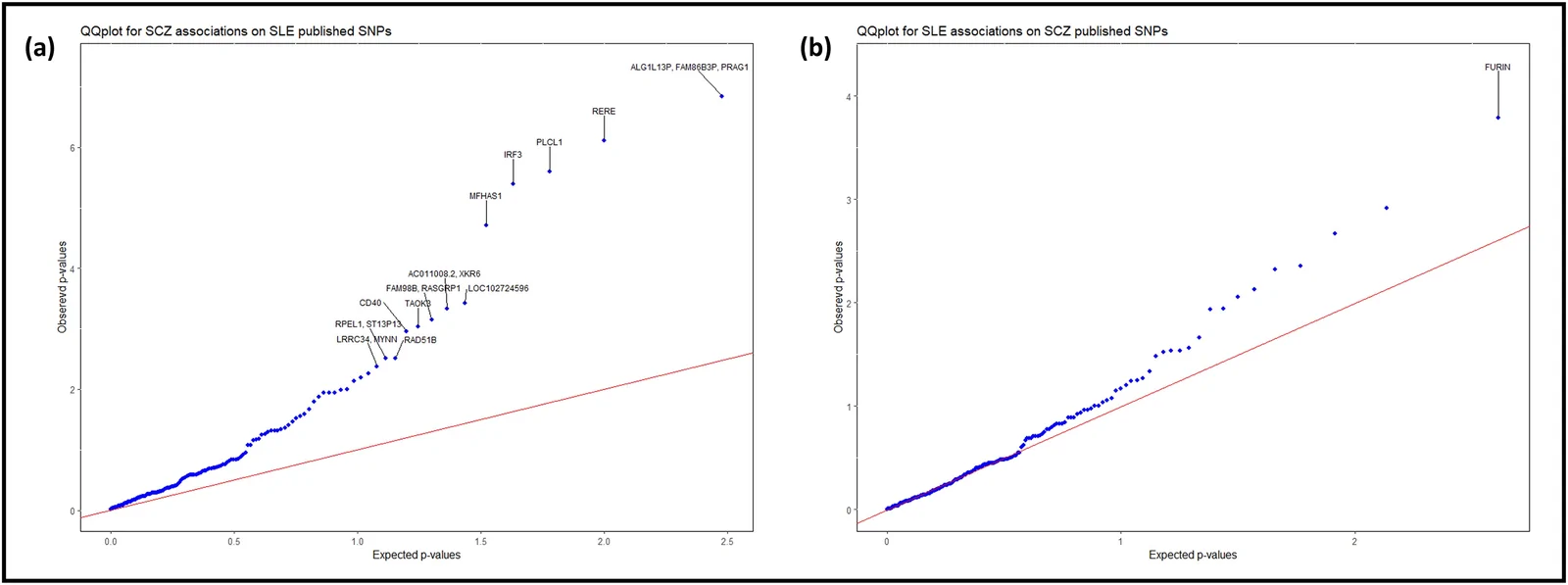
Quantile-quantile plots showing inflated genetic associations, when comparing SCZ and SLE. Q-Q plots display the distribution of associations with SLE for published SCZ SNPs and vice-versa (excluding MHC region). (a): Association in SCZ data for SNPs published as associated with SLE. (b): Association in SLE data for SNPs published as associated with SCZ.

To identify the genomic loci driving the global genetic correlation, we performed local genetic correlation analyses using ρ-HESS，including the extended MHC region [12]. We included an SLE Immunochip dataset (see methods) [10] to increase the power to detect local genetic correlations. We identified 16 loci with significant evidence of genetic correlation: 11 demonstrated positive correlation and five negative (Table 1). Two genome partitions in the extended MHC region demonstrated evidence of negative local correlation (p = 2.9 × 10^−9^/p = 9.1 × 10^−9^). The genome partition that contained the *FCGR2A* locus on chromosome 1q23 was the most significant and positively correlated (p = 3.8 × 10^−13^). We also observed significant positive local genetic correlations in a partition overlapping the *FURIN-FES* locus (p = 8.0 × 10^−6^), partitions near the *MFHAS1* locus (p = 1.0 × 10^−6^) and the *FAM98B-RASGRP1* locus (p = 1.3 × 10^−6^).

**Table 1:** Significant genome regions from ρ-HESS analysis. Significant genome partitions (p.adjust = 0.05/1690) are displayed, with column showing chromosome number (chr), genome partition start position (start), genome partition end position (end), number of SNPs in genome partitions (N_SNP_), number of eigenvectors used in truncated-SVD (k), local genetic covariance estimates (h_g(local)_), estimated variance of the local SNP-heritability (var) and standard error (se), z-score (z) and p-value (p). The ‘**Nearest Gene reported as associated with SCZ/SLE**’ are genes reported in publications for each trait. The ‘**Overlapped inflated SNPs**’ column shows SNPs associated in the Q-Q plot that are located within each genome partition. The associated trait is noted. The stars (*) at h_g(local)_ column identifies genome partitions that contain candidate shared loci from the cross-trait meta-analysis. The number of stars corresponds to the number of independent association signals identified in the meta-analysis.

**Significant genome regions from ρ-HESS analysis**												
**CHR**	**start (bp)**	**end (bp)**	**N** _**SNP**_	**k**	**h** _**g(local)**_	**var**	**se**	**Z-score**	**P-value**	**Nearest Genes reported as associated with SCZ**	**Nearest Genes reported as associated with SLE**	**Overlapped inflated SNPs**
1	159 913 048	162 346 721	3628	50	0.00108 *	2.21x10^−8^	0.000149	7.26	3.85x10^−13^	***INTS3,SLC27A3,LOC343052,*** ***GATAD2B,DENND4B,CRTC2***	***AL590385.2, FCGR2A, FCGR2C***	
6	32 682 664	33 236 497	78	8	−0.00125	4.43x10^−8^	0.000210	−5.94	2.93x10^−9^	***xMHC***	***ANKS1A, PPARD, UHRF1BP1***	
6	33 236 497	35 455 756	3228	50	−0.00124	4.64x10^−8^	0.000215	−5.75	9.05x10^−9^	***xMHC***	***ANKS1A, PPARD, UHRF1BP1***	
1	197 311 514	199 239 884	2039	50	0.000760	1.84x10^−8^	0.000136	5.60	2.16x10^−8^	***LINC00862***	***AL157402.1, PTPRC***	
8	11 278 998	13 491 775	3807	50	0.000972 *	3.58x10^−8^	0.000189	5.13	2.86x10^−7^	***MSRA***	***AF131216.5, BLK***	
16	68 841 363	71 054 028	1983	50	0.000764	2.40x10^−8^	0.000155	4.93	8.41x10^−7^	***CALB2, CMTR2, HYDIN***	***36P, RNU4, ZFP90***	
8	7 153 079	9 154 694	2356	50	0.000854 ***	3.06x10^−8^	0.000175	4.89	1.03x10^−6^	***FAM85B,FAM86B3P,PRAG1,*** ***CLDN23,MFHAS1,ERI1,***	***MFHAS1***	rs2955587 (SLE) rs2428 (SLE)
15	50 008 043	51 677 560	2420	50	0.000731	2.28x10^−8^	0.000151	4.83	1.32x10^−6^	***SEMA6D***	***FAM98B, RASGRP1***	
19	28 557 893	29 790 947	2940	50	−0.000620	1.70x10^−8^	0.000131	−4.79	1.69x10^−6^	***ZNF536***	***ANKRD27, PDCD5***	
16	13 154 437	14 464 002	2329	50	0.000792 *	3.05x10^−8^	0.000175	4.53	5.73x10^−6^	***ERCC4***	***CIITA, CLEC16A***	
15	90 475 551	92 164 392	2848	50	0.000638 *	2.04x10^−8^	0.000143	4.46	8.03x10^−6^	***FURIN,FES, FURIN, MAN2A2***	***LINC02253, RN7SKP181***	rs4702 (SCZ)
3	60 272 906	62 355 666	3711	50	−0.000650	2.31x10^−8^	0.000152	−4.29	1.82x10^−5^	***FHIT***	***AC098479.1, AC116036.2, PDHB, PXK***	
22	39 307 894	40 545 797	1500	50	0.000798 *	3.50x10^−8^	0.000187	4.26	2.03x10^−5^	***CACNA1I, LOC100506472, MGAT3, TAB1,*** ***RPS19BP1, MGAT3-AS1,***	***ENTHD1, GRAP2***	
6	134 244 243	136 224 177	2472	50	−0.000630	2.24x10^−8^	0.000150	−4.23	2.37x10^−5^	***SAMD3,TMEM200A***	***AL356234.2, AL591468.1, BTF3L4P3, LINC02528, TNFAIP3***	
6	162 931 203	164 383 521	2764	50	0.000607 *	2.08x10^−8^	0.000144	4.21	2.53x10^−5^	***RN7SL366P, C6orf118***	***IPCEF1***	
9	36 743 283	38 641 599	2976	50	0.000703	2.83x10^−8^	0.000168	4.18	2.94x10^−5^	***GNE,RNF38***	***IFNA22P***	

### Cross-trait meta-analysis

The evidence for SCZ-SLE genetic correlation prompted us to conduct a cross-trait meta-analysis using summary statistics from GWAS studies of both disorders, the same data used for the genome-wide genetic correlation analysis (see methods). This nominated 17 candidate loci with aligned effects and nine candidate loci with opposing effects that had genome-wide significant evidence (p < 5 × 10^−8^) of association in meta-analysis and nominal (p < 1 × 10^−2^) association in both diseases (Fig. 2, Table 2).

**Figure 2:**
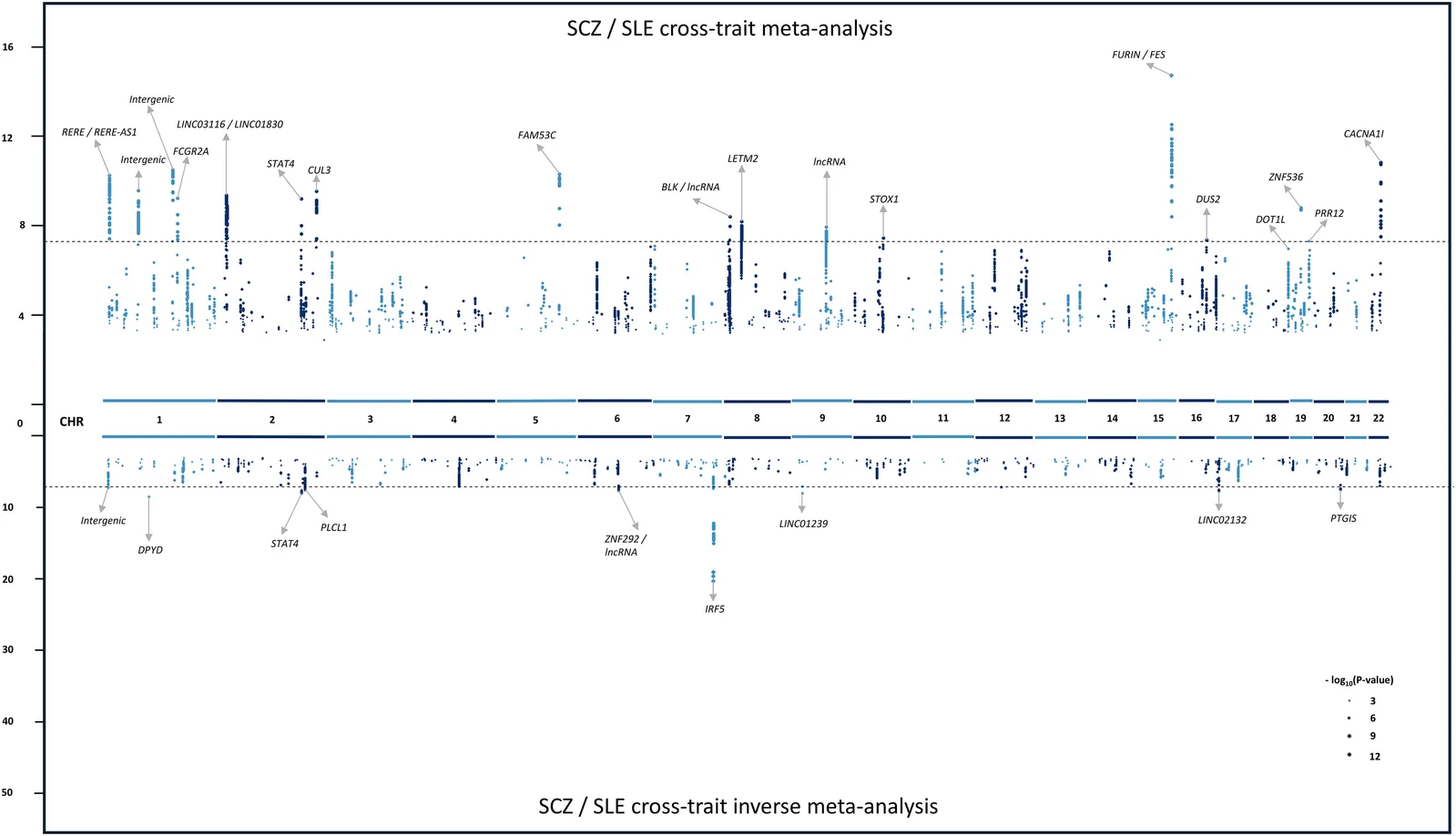
Manhattan plots of cross-trait meta-analysis associations. Manhattan plots showing the -log10 of the cross-trait meta-analysis p-values. Point size corresponds to the p-values, as shown in the legend. The upper plot shows the results from meta-analysis using a standard inverse-variance method, the lower plot shows the meta-analysis results when flipping the estimated effect size direction in SCZ data to detect opposing allelic effects. Signals that passed p-value < 5 × 10–8 (dotted line) were annotated according to variant effect predictor (VEP). The DOT1L locus was also annotated.

**Table 2:** Association results for lead SNPs in the cross-trait meta-analysis/inverse meta-analysis of SCZ and SLE data. The index variants of each candidate shared loci identified by cross-trait meta-analysis. The names of the loci are given by the most likely functional gene/transcript of index variant predicted by Ensembl variant effect predictor (VEP). The test statistics for lead associated variants in the cross-trait meta-analysis results are displayed. Novelty of loci in SCZ/SLE are also showed. The posterior probability (PP_H4_) of each candidate shared loci are also shown, the highest PP_H4_ are displayed if there were multiple independent signals at a locus. Loci with evidence of colocalisation are marked with asterisk (*) next to index SNP. Heterogeneity statistics from both the SLE multi-cohort meta-analysis and the SCZ-SLE cross-trait meta-analysis are reported in Supplementary Table 2.

**Associations with aligned allelic effects in cross-trait meta-analysis**													
**SNP**	**Position**		**Gene**	**Novel in SCZ?**	**Novel in SLE?**	**Effect allele**	**SCZ**		**SLE**		**Meta-analysis**		**SCZ – SLE coloc.PP**_**H4**_
	**CHR**	**BP** **(hg38)**					**Beta** **(s.e.)**	**Pval**	**Beta** **(s.e.)**	**Pval**	**Beta** **(s.e.)**	**Pval**	
rs301797	1	8 427 263	RERE/ RERE-AS1			A	0.068 (0.012)	2.7x10^−9^	0.068 (0.024)	5.2x10^−3^	0.068 (0.010)	5.6x10^−11^	33%
rs4300175	1	72 904 961	Intergenic			A	0.063 (0.011)	1.7x10^−8^	0.079 (0.027)	3.3x10^−3^	0.065 (0.010)	2.6x10^−10^	2.55%
rs72694965	1	150 186 473	Intergenic			A	−0.091 (0.015)	1.2x10^−9^	−0.091 (0.034)	7.0x10^−3^	−0.091 (0.014)	3.0x10^−11^	14.2%
rs10800314 *	1	161 502 999	FCGR2A	True		A	0.038 (0.011)	6.7x10^−4^	0.17 (0.023)	5.9x10^−13^	0.063 (0.010)	5.8x10^−10^	58.1%
rs13418451 *	2	22 427 316	LINC03116/LINC01830			T	−0.053 (0.011)	8.2x10^−7^	−0.096 (0.023)	2.9x10^−5^	−0.061 (0.0098)	4.2x10^−10^	69.9%
rs72913156	2	191 048 631	STAT4	True		A	0.029 (0.011)	7.0x10^−3^	0.19 (0.022)	5.5x10^−18^	0.061 (0.0098)	5.9x10^−10^	9.78%
rs11685299	2	224 526 579	CUL3		True	A	−0.065 (0.011)	1.1x10^−8^	−0.064 (0.024)	8.8x10^−3^	−0.065 (0.010)	2.9x10^−10^	20.7%
rs3756766	5	138 337 478	FAM53C		True	A	0.067 (0.012)	2.2x10^−8^	0.091 (0.026)	3.6x10^−4^	0.071 (0.011)	4.8x10^−11^	44.9%
rs2736332	8	11 482 456	BLK/lncRNA	True		C	0.032196 (0.012)	7.4x10^−3^	0.23 (0.027)	2.1x10^−17^	0.065 (0.011)	3.9x10^−9^	7.69%
rs112537273	8	38 390 788	LETM2		True	T	0.066 (0.013)	3.7x10^−7^	0.081 (0.028)	4.0x10^−3^	0.069 (0.012)	6.2x10^−9^	34.8%
rs6559666	9	82 447 144	lncRNA			T	0.056 (0.012)	4.0x10^−6^	0.098 (0.027)	2.7x10^−4^	0.063 (0.011)	1.1x10^−8^	5.31%
rs72801529	10	68 863 928	STOX1	True	True	T	0.051 (0.012)	1.9x10^−5^	0.092 (0.024)	1.4x10^−4^	0.059 (0.011)	3.5x10^−8^	25.3%
rs4702 *	15	90 883 330	FURIN/FES		True	A	−0.078 (0.011)	2.3x10^−12^	−0.083 (0.022)	1.6x10^−4^	−0.079 (0.010)	1.9x10^−15^	86.6%
rs1985437 *	16	68 040 954	DUS2		True	A	−0.062 (0.013)	1.0x10^−6^	−0.074 (0.029)	9.0x10^−3^	−0.064 (0.012)	4.2x10^−8^	6.08%
rs141958336 *	19	2 165 384	DOT1L			A	0.11 (0.025)	2.0x10^−5^	0.21 (0.058)	3.8x10^−4^	0.12 (0.023)	1.1x10^−7^	65.9%
rs1469705	19	30 548 088	ZNF536		True	T	−0.063 (0.012)	8.4x10^−8^	−0.070 (0.025)	5.0x10^−3^	−0.064 (0.011)	1.5x10^−9^	10.5%
rs6509439	19	49 601 982	PRR12		True	T	−0.055 (0.012)	5.0x10^−6^	−0.074 (0.024)	2.3x10^−3^	−0.059 (0.011)	4.9x10^−8^	11.4%
rs132582 *	22	39 592 170	CACNA1I		True	T	−0.067 (0.011)	5.1x10^−10^	−0.061 (0.022)	6.6x10^−3^	−0.066 (0.0097)	1.5x10^−11^	6.12%
**SNP**	**Position**		**Gene** **(Predicted by VEP)**	**Novel in SCZ?**	**Novel in SLE?**	**Effect allele**	**SCZ**		**SLE**		**Meta-analysis**		**SCZ- SLE coloc.PP**_**H4**_
	**CHR**	**BP**					**Beta** **(s.e.)**	**Pval**	**Beta** **(s.e.)**	**Pval**	**Beta** **(s.e.)**	**Pval**	
rs10864633	1	6 713 202	intergenic			A	−0.046 (0.011)	1.9x10^−5^	0.085 (0.023)	2.0x10^−4^	0.053 (0.0098)	4.7x10^−8^	29.6%
rs11165922	1	97 871 267	DPYD		True	T	−0.058 (0.011)	1.2x10^−7^	0.060 (0.023)	7.8x10^−3^	0.059 (0.0099)	2.7x10^−9^	2%
rs13407419	2	191 103 694	STAT4	True		T	−0.057 (0.019)	2.4x10^−3^	0.28 (0.040)	1.4x10^−12^	0.098 (0.017)	7.8x10^−9^	9.79%
rs10497813	2	198 049 348	PLCL1			T	−0.052 (0.011)	1.0x10^−6^	0.069 (0.025)	4.8x10^−3^	0.054 (0.0097)	2.2x10^−8^	4.54%
rs9767370	6	87 150 621	ZNF292/lncRNA	True	True	T	0.11 (0.023)	1.0x10^−6^	−0.15 (0.049)	3.0x10^−3^	−0.12 (0.021)	1.7x10^−8^	34.6%
rs4731532	7	128 932 712	IRF5	True		A	−0.033 (0.011)	2.5x10^−3^	0.34 (0.022)	1.2x10^−52^	0.092 (0.0098)	4.8x10^−21^	13.2%
rs9695226	9	22 759 397	LINC01239			A	−0.059 (0.012)	2.5x10^−7^	0.064 (0.024)	8.2x10^−3^	0.060 (0.010)	6.9x10^−9^	36.4%
rs305066	16	85 939 170	LINC02132			T	−0.036 (0.012)	1.8x10^−3^	0.16 (0.024)	8.1x10^−11^	0.059 (0.010)	1.6x10^−8^	2.19%
rs546674	20	49 501 461	PTGIS		True	T	−0.056 (0.012)	2.0x10^−6^	0.070 (0.025)	4.0x10^−3^	0.059 (0.011)	2.5x10^−8^	6.06%

Among the 17 loci with aligned effects, five have lead SNPs that reside within the significantly correlated genome partitions, including *FCGR2A* on chromosome 1, *BLK* locus on chromosome 8, *FURIN-FES* on chromosome 15, *DUS2* locus on chromosome 16 and *CACNA1I* on chromosome 22. For the nine candidate associations in the cross-trait meta-analysis with opposing effects, none reside within the significantly correlated genome partitions (Table 1, Table 2). The *FURIN-FES* locus on chromosome 15, with index SNP rs4702, a known SCZ-associated locus [6], showed the most significant meta-analysis p-value with the same direction of effect for both diseases. A subset of nominated candidate loci showed evidence of cross-trait heterogeneity (Supplementary Table 2), whereas the *FURIN-FES* locus, which was prioritised for detailed follow-up analyses, did not.

To assess whether identified shared loci converged on common biological processes, we performed pathway enrichment analysis using genes mapped from the 26 candidate shared loci identified in the cross-trait meta-analysis using the STRING database [13]. The mapped genes highlighted association with autoimmune-related diseases (Supplementary Table 3). However, we did not observe significant enrichment for neuronal pathways or for a unified biological process. The lack of evidence for common biological pathways prompted us to evaluate whether individual loci reflected shared causal variants rather than overlapping regional association, and to investigate their functional consequences in downstream analyses.

We performed colocalisation analysis on all candidate shared loci from the cross-trait meta-analysis to assess whether the two diseases’ association signals were due to a shared causal variant rather than coincidental overlap. This provided strong evidence of colocalisation at the *FURIN-FES* locus, a locus consistently identified through inflated associations, local genetic correlation analysis and cross-trait meta-analysis (Fig. 3). The posterior probability of SCZ and SLE sharing the same causal SNP at *FURIN-FES* was 86.6% (Fig. 4). Notably, rs4702 was identified as the only candidate shared causal SNP (SNP.PP_H4_ = 96.3%). We also identified two additional loci with evidence of colocalisation; a locus within intergenic noncoding RNA (*LINC03116*/*LINC01830*) (coloc.PP_H4_ = 69.9%), and the *FCGR2A* locus (coloc.PP_H4_ = 58.1%) (Supplementary Fig. 1). We did not find evidence of colocalisation for any of the loci with significant meta-analysis -association that had opposing allelic effects across the two diseases (Table 2).

**Figure 3:**
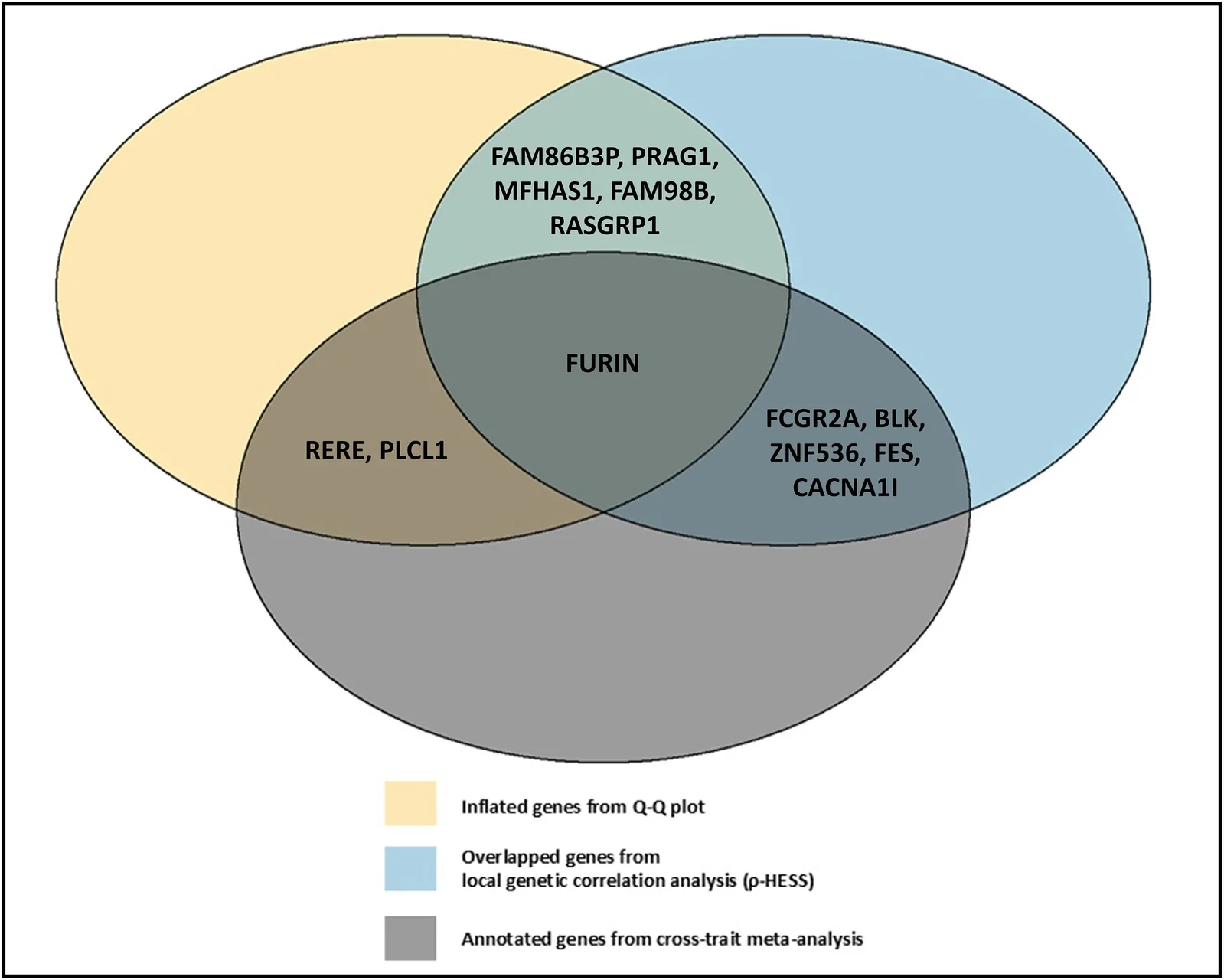
Venn diagram illustrating the overlap of loci identified in the three main analyses to test for ‘sharing’. The diagram shows the overlap of genes among three gene sets: Genes identified by inflated associations in the other trait GWAS (upper-left circle), genes from genome partitions showing significant correlation (upper-right circle), and genes from significant loci in the cross-trait meta-analysis (lower circle).

**Figure 4:**
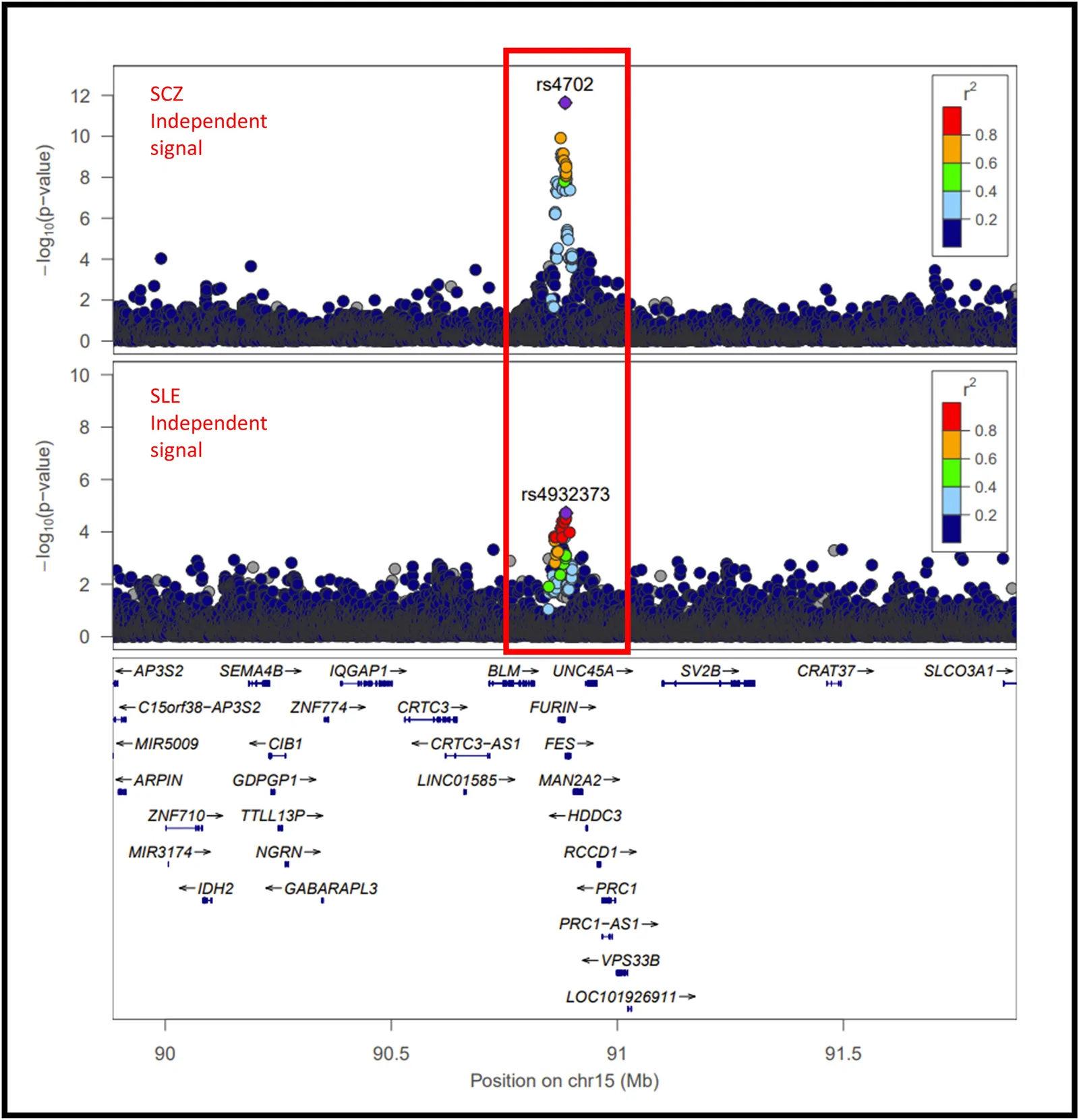
LocusZoom plots at FURIN-FES locus for association with SCZ and SLE. LocusZoom plots at the FURIN-FES locus (highlighted by an outlined rectangle) in SCZ and SLE data. The lead association SNP in each data is the LD index SNP.

In addition to above candidate shared loci, we also found moderate evidence of colocalisation (coloc.PP_H4_ = 65.9%) (Supplementary Fig. 2) at the *DOT1L* locus on chromosome 19, a locus with close to genome-wide level of significance at meta-analysis (cross-trait meta p-value = 1.07 × 10^−7^).

### Effects of SCZ-SLE shared loci on gene expression

To investigate whether loci identified as having shared genetic association impact function through altered gene expression, we assessed expression quantitative trait loci (eQTLs) using data from eQTLGen (Phase I) whole blood data [14], 25 single-tissue datasets in European-American subjects from GTEx v8 [15] (see Supplementary Table 4 for detailed tissue types), 11 blood immune cell types in the eQTL catalogue [16] (see Supplementary Table 5 for details) and eight major cell types in the brain [17]. We focused only on loci that had evidence (PP_H4_ > 50%) in support of colocalisation between SCZ/SLE associations which included *FCGR2A*, *LINC03116*/*LINC01830*, *FURIN-FES* and *DOT1L*. For each of the loci, and for both diseases, we checked all independent association signals for colocalisation with independent eQTL signals.

### FURIN-FES locus

The SNPs leading the SCZ-SLE shared association signals at *FURIN-FES* were significant *cis*-eQTLs for multiple genes across the eQTL datasets. The eGenes we identified include *FURIN*, *FES*, *MAN2A2*, *RCCD1* and *HDDC3*. We checked for evidence of colocalisation between SCZ/SLE GWAS signals and *cis*-eQTL of the above eGenes in whole blood data of eQTLGen. There was a strong *FURIN cis*-eQTL signal in the whole blood eQTLGen data at the *FURIN-FES* locus near the SCZ and SLE signal, but we did not find evidence of colocalisation (Supplementary Fig. 3). In the eQTLGen data *trans*-eQTL analysis, we identified three candidate *trans*-eGenes (*SPARC*, *CTTN* and *ACER3*) associated with a variant (rs2521501) that is in high LD with the SLE lead SNP (r^2^ = 0.95) and in moderate LD with the SCZ lead SNP (r^2^ = 0.34). However, we were unable to confirm whether the SCZ, SLE, and eQTL signals for these *trans*-eGenes share a common causal variant at the *FURIN-FES* locus, primarily due to limited SNP coverage in the eQTLGen *trans*-eQTL dataset (only 9 SNPs, compared to > 4000 in *cis*-eQTL dataset typically available for colocalisation analysis).

In the GTEx v8 data, there was good evidence supporting colocalisation between the SCZ/SLE GWAS signals at the *FURIN-FES* locus and *FURIN cis*-eQTL signal in tissue sampled from the ascending aorta (SCZ.coloc.PP_H4_ = 99.9%, SLE.coloc.PP_H4_ = 83.1%) (Fig. 5a). rs4702 was the most likely shared causal SNP for the colocalisation with SCZ, and in LD (r^2^ > 0.6) with the most likely causal SNP (rs8039305) for the colocalisation with SLE. The G allele of the most likely shared causal SNP rs4702, that was associated with increased risk in both SCZ and SLE, was associated with increased *FURIN* expression in this artery (Supplementary Table 6). We did not find evidence supporting colocalisation between the SCZ/SLE association signals at the *FURIN-FES* locus and *cis*-eQTLs of the other eGenes in tested tissues. No additional *trans*-eQTLs were detected among all tissues in GTEx, consistent with the limited number of significant *trans*-eGenes reported in that dataset.

**Figure 5:**
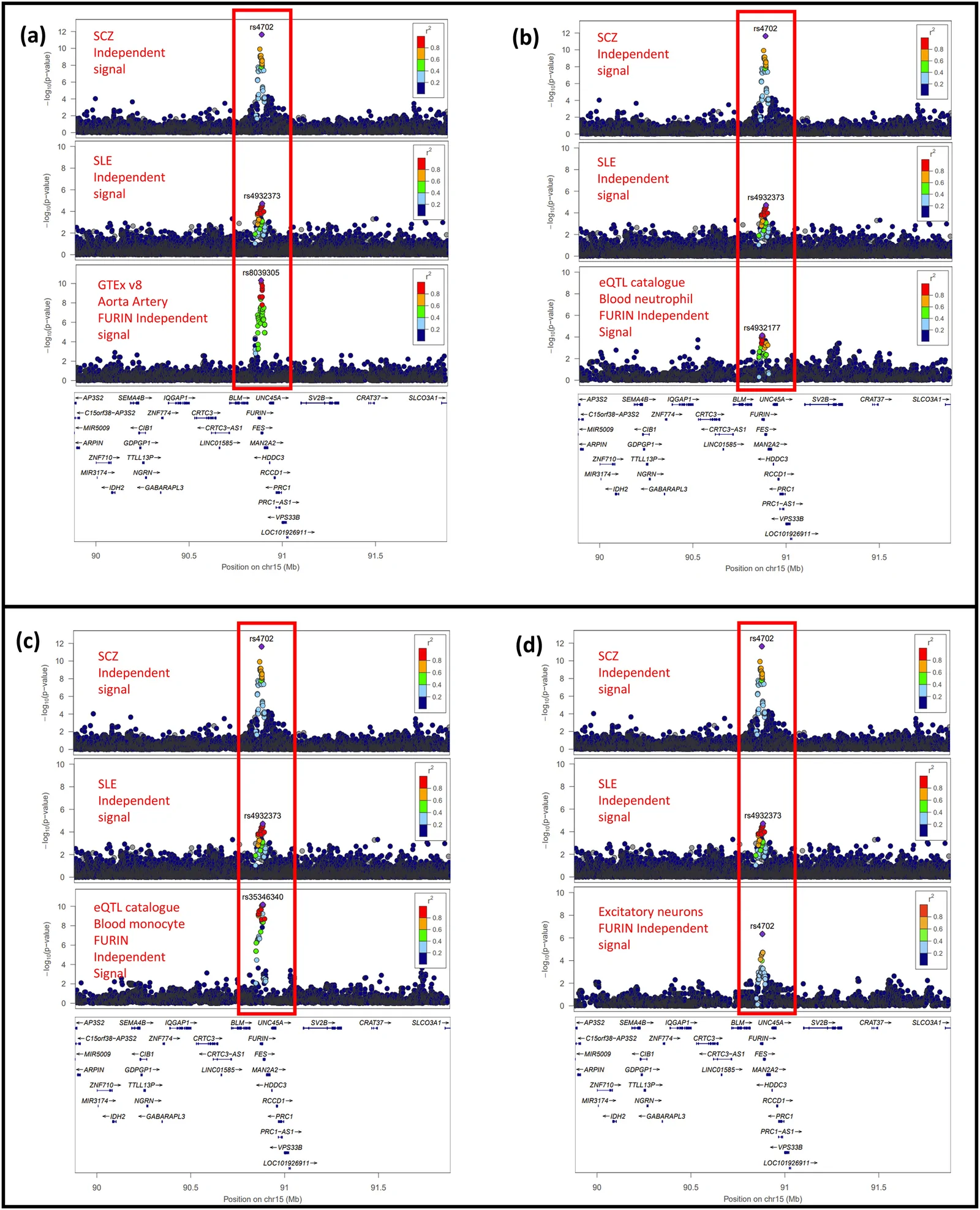
LocusZoom plots at the FURIN-FES locus for association with SCZ, SLE and FURIN eQTL. LocusZoom plots at the FURIN-FES locus (highlighted by an outlined rectangle) with regional association signals. In each plot from top to bottom are the SCZ signal, SLE signal and FURIN cis-eQTL signal (a) SCZ/SLE/FURIN cis-eQTL from aorta artery of GTEx (b) SCZ/SLE/FURIN cis-eQTL from isolated naïve neutrophil data (c) SCZ/SLE/FURIN cis-eQTL from isolated naïve monocyte data (d) SCZ/SLE/FURIN cis-eQTL from excitatory neurons of brain.

To investigate which immune cell types were contributing to the eQTL signals that colocalised with both diseases, we examined *FURIN* and *FES cis*-eQTL data from studies of isolated human immune cell types deposited on the eQTL catalogue [16] including data sampled from major immune cells of myeloid and lymphoid lineages (see Methods). We found that the shared SCZ/SLE independent signal colocalized with a *FURIN cis*-eQTL signal in naïve monocytes (SCZ.coloc.PP_H4_ = 94.3%, SLE.coloc.PP_H4_ = 96.6%) and a *FURIN cis*-eQTL signal in neutrophils (SCZ.coloc.PP_H4_ = 73.6%, SLE.coloc.PP_H4_ = 59.2%) isolated from whole blood (Fig. 5b and c) [18]. For all colocalisations, rs4702 was the most likely causal SNP for the colocalisation with SCZ, and in weak LD (r^2^ = 0.35) with the most likely causal SNP (rs4932373) for SLE, the risk allele for the SCZ-SLE shared association was associated with decreased expression of FURIN in both these cell types.

We also found strong evidence that the SCZ-SLE shared association signals colocalized with a *FES cis*-eQTL, a gene very close to *FURIN*, in multiple immune cell types, including naïve state macrophage (SCZ.coloc.PP_H4_ = 69.8%,SLE.coloc.PP_H4_ = 87%), naïve state lymphoblastoid cell line (LCL) (SCZ.coloc.PP_H4_ = 52.1%, SLE.coloc.PP_H4_ = 95.3%), blood monocyte treated with IFN-γ and lipopolysaccharide (LPS) (SCZ.coloc.PP_H4_ = 81.1%, SLE.coloc.PP_H4_ = 50.2%) and isolated naïve state T cells from healthy new born umbilical cord (SCZ.coloc.PP_H4_ = 58%, SLE.coloc.PP_H4_ = 95.8%) (Fig. 6a-d). For naïve state macrophage, LCL and T cells, rs4702 was the most likely causal SNP for the coloc with SCZ, and in weak LD (r^2^ = 0.36) with the most likely causal SNP (rs12906125/rs35346340) for the coloc with SLE. For monocyte received IFN-γ and LPS treatment, rs4702 was in moderate LD (r^2^ = 0.69) with the most likely causal SNP (rs8039305) for the colocalisation with SLE and SCZ. In all immune cells mentioned above, the disease risk allele was associated with increased *FES* expression (Supplementary Table 6).

**Figure 6:**
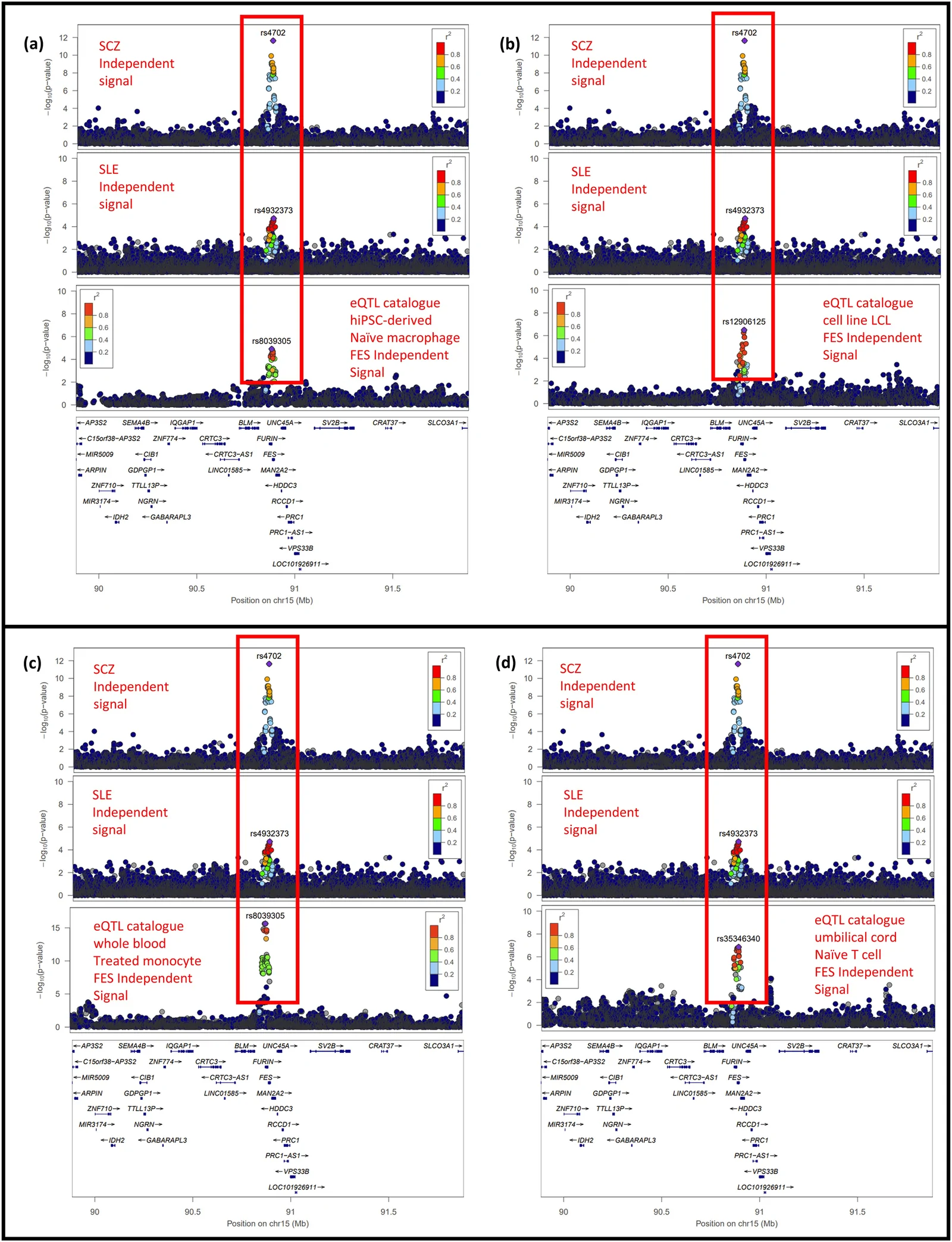
LocusZoom plots at the FURIN-FES locus for association with SCZ, SLE and FES eQTL. LocusZoom plots at the FURIN-FES locus (highlighted by an outlined rectangle) with regional association signals. In each plot from top to bottom are the SCZ/SLE and FES cis-eQTL signals. (a) SCZ/SLE/FES cis-eQTL from human iPSCs-derived macrophage. (b) SCZ/SLE/FES cis-eQTL from naïve lymphoblastoid cell lines (c) SCZ/SLE/FES cis-eQTL from whole blood—Isolated monocyte treated with IFN-γ + 24 hr lipopolysaccharide. (d) SCZ/SLE/FES cis-eQTL from naïve T cells isolated from healthy newborn umbilical cord.

Our analyses of cis-eQTL data from eight major human brain cell types (see Methods) [17] included oligodendrocyte precursor cells (OPCs), inhibitory/excitatory neurons, pericytes, endothelial cells, microglia, astrocytes and oligodendrocytes. We found that only a *FURIN cis*-eQTL signal in excitatory neurons of the brain colocalized with the shared SCZ and SLE signal (SCZ.coloc.PP_H4_ = 99.9%, SLE.coloc.PP_H4_ = 83.1%) (Fig. 5d). rs4702 was the most likely causal SNP, with the disease risk allele associated with decreased *FURIN* expression in brain excitatory neurons (Supplementary Table 6). We did not find evidence supporting colocalisation with *FES cis*-eQTLs in all tested brain cells.

### Other disease association shared loci and eQTL colocalisation

At the *DOT1L* locus, the SCZ/SLE shared association signals were significant cis-eQTLs for *DOT1L*, *AMH*, *AP3D1*, *SF3A2*, *PLEKHJ1* (Supplementary Table 4). We find evidence of colocalisation between the SCZ, SLE signals and *DOT1L* in Transverse Colon only and with *PLEKHJ1* in multiple tissues (see **Supplementary Results**, Supplementary Fig. 4 and 5 for details). The SCZ-SLE shared association signal at *FCGR2A* was also a significant *cis*-eQTL for multiple genes in whole blood data, including three genes encoding Fc gamma receptors and heat shock proteins (Supplementary Table 4). However, no evidence of colocalisation was found between the SCZ-SLE shared association signal and the identified eGenes across all tested eQTL datasets. For the SCZ-SLE shared association signal at the long intergenic non-coding RNA (*LINC03116*/*LINC01830*) region on chromosome 2, no significant eQTL was identified across all tested eQTL datasets.

### Functional effects of SCZ-SLE shared loci on protein expression

To explore the effects of SCZ-SLE shared association signals on protein levels, we identified cis and trans protein quantitative trait loci (pQTL) at disease associated loci using data from plasma [19, 20],brain [21] and CSF [22] (see Methods for the data source details). For every pQTL observed, we tested for colocalisation. Due to the biological action of FURIN protein, we further tested for colocalisation between disease association and pQTL signals for all genes known to interact with FURIN from previous studies, as detailed in Methods.

In the plasma proteomics data from the UK biobank, we found the SCZ-SLE shared association signals at the *FURIN-FES* locus had strong evidence of colocalisation with *cis*-pQTLs for FURIN protein (SCZ.coloc.PP_H4_ = 59.6%, SLE.coloc.PP_H4_ = 95.4%) (Fig. 7a), and evidence of colocalisation with *cis*-pQTL for the tyrosine kinase FES (SCZ.coloc.PP_H4_ = 51.8%, SLE.coloc.PP_H4_ = 53.9%) (Fig. 7b). The disease risk allele was associated with decreased FURIN level but increased FES level in plasma (Supplementary Table 7).

**Figure 7:**
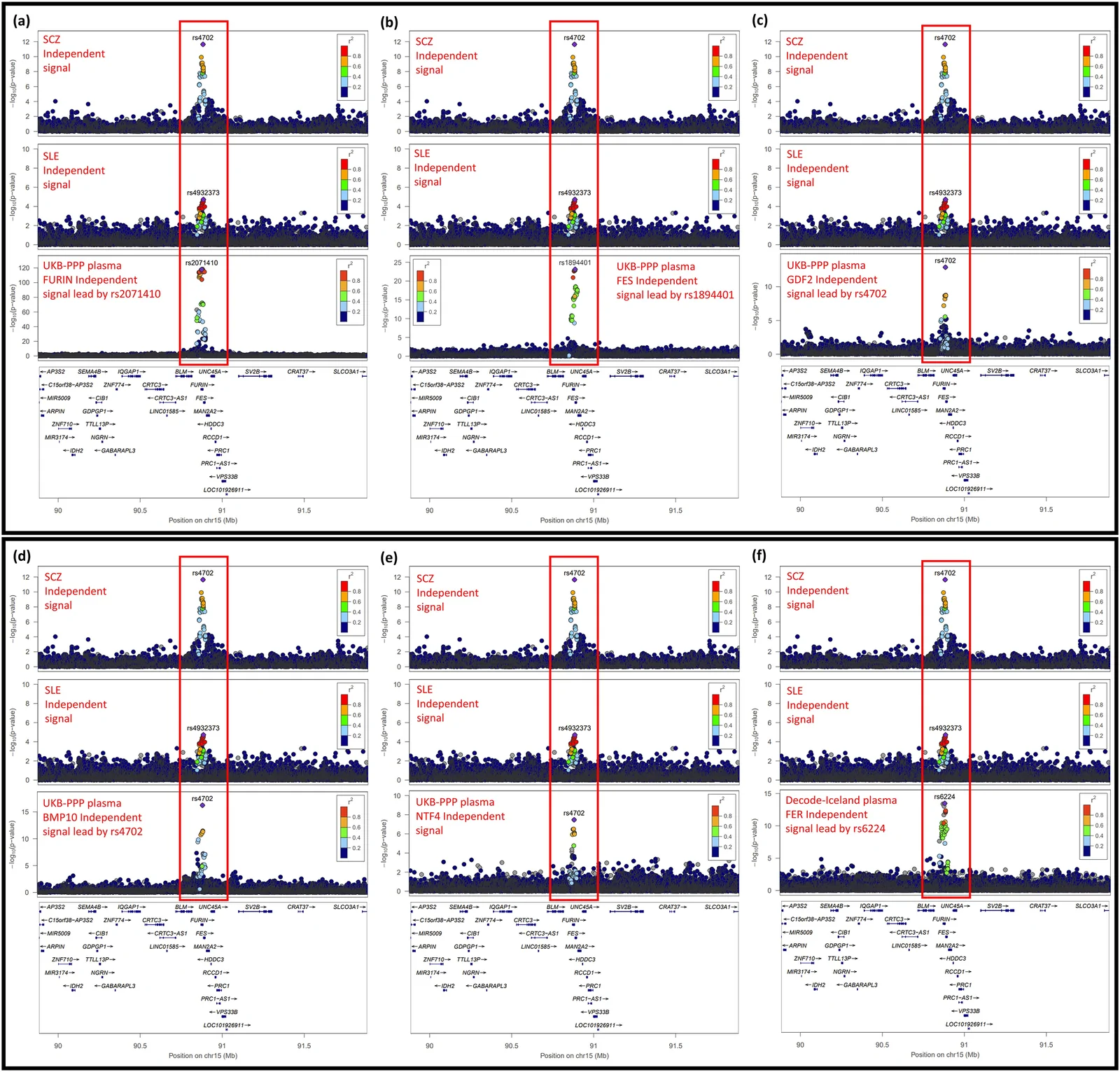
LocusZoom plots at the FURIN-FES locus for association with SCZ, SLE and pQTL. LocusZoom plots at the FURIN-FES locus (highlighted by an outlined rectangle) with regional association signals. In each plot from top to bottom are the SCZ signal, SLE signal and pQTL signal. (a) SCZ/SLE/plasma Furin cis-pQTL. (b) SCZ/SLE/marginal association of plasma Fes cis-pQTL lead by rs1894401. (c) SCZ/SLE/plasma GDF2 transpQTL. (d) SCZ/SLE/plasma BMP10 trans-pQTL. (e) SCZ/SLE/plasma NTF4 trans-pQTL. (f) SCZ/SLE/marginal association of plasma fer trans-pQTL lead by rs6224.

We also identified evidence of colocalisation in the *FURIN-FES* locus with *trans*-pQTL for known targets of FURIN, including TGF-β family members: GDF2 (SCZ.coloc.PP_H4_ = 100%, SLE.coloc.PP_H4_ = 85.1%) and BMP10 (SCZ.coloc.PP_H4_ = 100%, SLE.coloc.PP_H4_ = 85.1%), and also *trans*-pQTL for a neurotrophin: NTF4 (SCZ.coloc.PP_H4_ = 99.9%, SLE.coloc.PP_H4_ = 79.9%). LocusZoom plots for these disease-pQTL associations can be seen in Fig. 7c-e. The disease risk allele was associated with decreased BMP10, GDF2, NTF4 protein level in plasma (Supplementary Table 7).

In the plasma proteomics data from Icelanders in the DECODE project, we found moderate evidence of colocalisation with *trans*-pQTL for SNPs in the *FURIN-FES* locus with FER (FES-related), another tyrosine kinase structurally related to FES (SCZ: PP_H4_ = 77.2%, SLE: PP_H4_ = 50.2%), see Fig. 7f. The disease risk allele associated with increased FER protein (Supplementary Table 7).

We did not find evidence of colocalisation between SCZ-SLE shared association signals outside of *FURIN-FES* and plasma proteomics data, and no colocalisation between pQTL signals in brain/cerebrospinal fluid (CSF) and the SCZ-SLE shared association signals.

### Causative inference of SCZ-SLE shared loci using MR

To complement the colocalisation analysis and assess potential causal relationships between proteins identified in the pQTL analysis and disease risk, we performed bi-directional, two-sample Mendelian randomization (MR) [23] on all colocalized pQTL-GWAS locus pairs (complete MR results on Supplementary Table 8). Overall, we observed strong evidence for causal relationships between FES plasma protein levels and diseases in forward MR (protein to diseases) analysis.

Using plasma protein data from the UK Biobank, we found evidence that FES protein levels have a positive causal relationship with both SCZ (Supplementary Fig. 6a) and SLE (Supplementary Fig. 6b) based on the inverse-variance weighted (IVW) method, with odds ratios of 1.80 (p-value = 2.91 × 10^−7^) and 2.61 (p-value = 5.64 × 10^−3^), respectively. In contrast, similar analyses of the FURIN protein, which was also a *cis*-pQTL, provided no evidence supporting a causal relationship with SCZ and SLE in either direction. For proteins with *trans*-pQTL effects (GDF2, BMP10, NTF4, FER), no significant causal effects were detected in either direction, though these analyses were constrained by the absence of valid IVs for several MR tests (Supplementary Table 8). The results suggest that the FES protein may directly influence the risk of both SCZ and SLE with aligned effect direction.

## Discussion

Our study provides a well-powered genome-wide comparison of schizophrenia (SCZ) and systemic lupus erythematosus (SLE), with the largest European SLE cohort to date [7–10]. We demonstrate a shared genetic architecture between SCZ and SLE, which would appear to support the hypothesis of shared genetic risk factors outside the MHC region that contribute to the pathogenesis of SCZ and SLE, but through different biological pathways. Our results caution against the over-interpretation of correlation between genetic association datasets as shared function. It is clear that detailed in silico and laboratory-based analysis and experimentation are required to conclude shared mechanism from apparent shared association.

### Shared genetic risk and functional insights

We observed a positive genome-wide genetic correlation between SCZ and SLE, consistent with previous studies [5]. Our higher genetic correlation estimate likely arises from the increased power and SNP density of the SLE data. We further identified multiple lines of evidence supporting local genetic overlap between SCZ and SLE. Local genetic correlation analysis further identified 16 genomic regions contributing to this overlap, with 11 positive and 5 negative correlations. Cross-trait meta-analysis nominated 26 candidate loci with genome-wide significant combined evidence and nominal association in both traits, and was used as a discovery approach to prioritise loci for subsequent colocalisation and functional follow-up analyses. We note that some nominated loci showed evidence of heterogeneity between SCZ and SLE effect estimates (Supplementary Table 2), which is not unexpected given the distinct biology of the two diseases. For this reason, candidate loci identified through meta-analysis were not interpreted as evidence of shared causal mechanisms in isolation, but were considered alongside local genetic correlation and subsequently evaluated using colocalisation, eQTL/pQTL analyses and Mendelian randomisation.

Pathway enrichment analysis of the candidate shared loci did not identify a common biological pathway or neuropsychiatric terms. This may partly reflect imbalances in database representation, as immune-related pathways are generally better annotated than neuronal ones. Also, many of the input genes have established immune functions, while relatively few are linked to neuronal processes.

Consistent with prior work, the extended MHC region exhibited negative genetic correlation (Table 1), reflecting opposing effects. This aligns with prior studies showing reciprocal genetic effects at the *C4* locus due to copy number variation [4]. The identified candidate shared loci outside the MHC region include potential novel association for SCZ, such as *FCGR2A* and *BLK*, and potential novel associations for SLE, such as *FURIN-FES* and *CACNA1I* (Table 2, Supplementary Table 1). Notably, 12 loci were consistently identified through more than one approach (Fig. 3).

Although we identified polygenic overlap at multiple regions across the genome, the evidence for shared causal variants among candidate shared loci was limited. Only a small subset of loci showed evidence consistent with colocalisation, most notably *FURIN-FES*, with more modest support at *DOT1L*, *FCGR2A*, and a locus within long intergenic non-coding RNA (*LINC03116/LINC01830*). The absence of colocalisation suggests that most regional genetic correlations between SCZ and SLE are driven by distinct causal variants located in proximity, rather than true pleiotropy of same causal variant. These results proposed a model in which SCZ-SLE genetic overlap reflects locus-specific and mechanistically distinct processes, rather than a coherent shared pathway.

### FURIN-FES locus: Pleiotropy via distinct pathways

The *FURIN-FES* locus on chromosome 15 provides the strongest evidence for a shared genetic signal between SCZ and SLE, and was previously implicated in SCZ but novel for SLE [6]. This locus was consistently identified across all analytical approaches (Fig. 3). It also represents the only locus with robust support for a shared causal variant.

The variant rs4702 emerged as the most likely shared causal SNP and exerts distinct regulatory effects on neighbouring genes. The disease risk allele is associated with reduced *FURIN* expression in neuronal and immune cells, and increased *FES* expression in immune cells, indicating gene- and cell-type-specific regulation. Integration of pQTL and MR analyses further indicates divergent downstream consequences. While shared disease association signals at *FURIN-FES* colocalised with *trans*-acting pQTLs for NTF4, BMP10, and GDF2, there is no evidence supporting a direct causal role for these or FURIN protein levels in either disease (see Fig. 8, **Supplementary Results**, Supplementary Fig. 7 and 8 for detailed hypothetical mechanisms). In contrast, FES plasma protein levels show evidence of a causal relationship with both SCZ and SLE, but the underlying biological mechanisms remain unclear based on current understanding of FES, and our results do not support a single unified pathway to both diseases. (see Fig. 8, **Supplementary Results**, Supplementary Fig. 9 and 10 for detailed hypothetical mechanisms).

**Figure 8:**
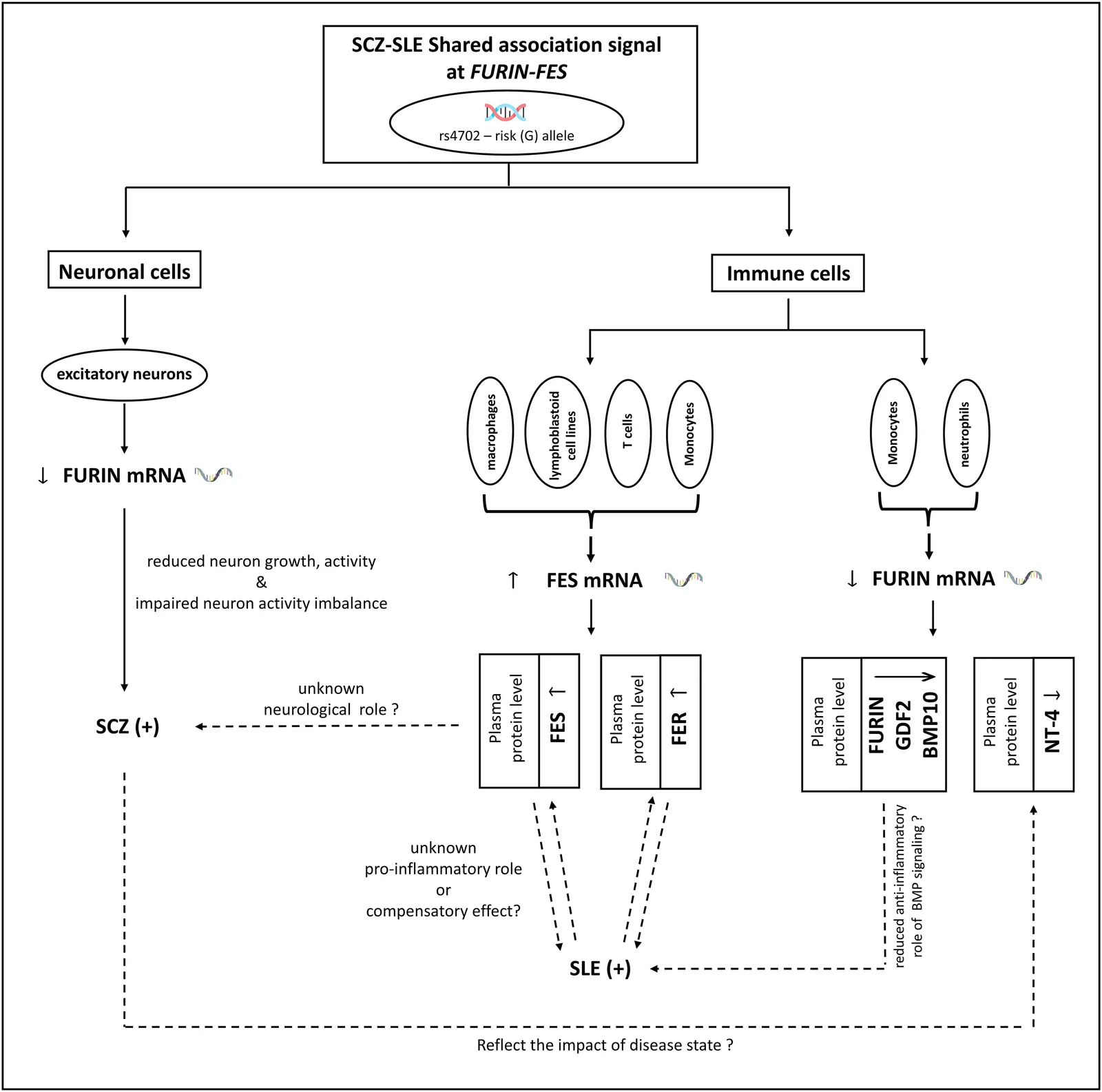
Functional impacts of the shared association signal at FURIN-FES and proposed biological mechanisms relevant to diseases. The plot illustrates the functional impact of the SCZ-SLE shared association signal at the FURIN-FES locus. Dashed lines are used to represent hypothetical mechanisms.

These findings support a dual-gene regulatory model, in which a single variant influences multiple genes with distinct functional contexts. One interpretation is that this locus contributes to SCZ risk through neuronal mechanisms mediated by *FURIN*, and to SLE through immune-related pathways involving both *FURIN* and *FES*. An alternative explanation is that the shared genetic signal may also reflect neurological involvement in SLE, which is consistent with the neuropsychiatric manifestations of the disease. Under this model, altered *FURIN* expression in neuronal tissues could contribute to neurological features in SLE.

### Other candidate shared loci

The *DOT1L* locus demonstrated moderate evidence of a shared causal variant. This locus has been recently identified to be associated with SLE [24], and SCZ [25]. Our functional assessment revealed that the shared signal was associated with altered *DOT1L* expression in a limited tissue context and broader effects on *PLEKHJ1* expression across multiple tissues, but no effects in immune or neuronal cell types. These findings indicate regulatory complexity involving *DOT1L* and *PLEKHJ1* at this locus, without clear evidence for a mechanism linking both diseases.

The *FCGR2A* locus showed strong local genetic correlation and moderate evidence of colocalisation. *FCGR2A* is a well-established susceptibility locus for SLE [7], but is a novel signal in SCZ. The functional interpretation at this locus is complicated by structural variation, which limits resolution of causal relationships between genetic association and gene expression. Although the locus maps to genes encoding Fc gamma receptors (*FCGR2A*, *FCGR2C*, and *FCGR3B*) as well as heat-shock proteins family A members (Supplementary Table 4), the presence of structural variation precludes further inference of causality.

The intergenic *LINC03116/LINC01830* locus showed evidence of colocalisation, but lacks detectable functional consequences in current datasets.

Beyond the *FURIN-FES* locus, the broader set of candidate shared loci suggests that the overlap between SCZ and SLE is biologically mixed rather than centred on a single common mechanism. Several loci with aligned effects, including *FCGR2A*, *BLK*, *STAT4* and *IRF5*, map to well-established immune pathways and are more readily interpreted in the context of SLE or general immune regulation, whereas loci such as *CACNA1I*, *CUL3*, *RERE*, *ZNF536*, and *PRR12* are more consistent with neurodevelopmental or brain-related biology. This pattern argues against a simple model in which the SCZ-SLE correlation is driven by one shared immune programme. Instead, it is more compatible with a combination of immune-weighted loci, brain-weighted loci, and a smaller number of genuinely pleiotropic regions. Within this framework, *FURIN-FES* stands out as the strongest shared locus and is also notable because it appears to be novel for SLE. Importantly, even at this locus, our functional data support context-specific effects rather than a single convergent downstream pathway.

### Limitations of research and future perspectives

Several limitations should be acknowledged for our study. Firstly, our analyses were primarily conducted in European populations, which may limit generalizability. Future studies in other populations are required to determine whether the observed shared genetic associations patterns are consistent across ancestries. This is particularly important for replicating the novel SLE association at the *FURIN-FES* locus in other independent GWAS datasets. We are aware of results from a large East Asian-European SLE meta-analysis (personal communication, East Asian-European SLE Meta-analysis Consortium) showing that the association at the *FURIN-FES* locus replicates robustly in East Asian ancestry. We also replicated this association in the recent Pan-UK Biobank genome-wide association analysis [26], where rs4702 showed significant associations (one-sided test; risk allele = G) with non-systemic lupus affecting the skin (p = 0.0005; 170 cases vs. 411 160 controls; phenotype code 695.41) and with non-systemic lupus affecting the cutaneous and subcutaneous tissue (p = 0.0004; 166 cases vs. 420 365 controls; phenotype code L93) in European populations. This variant also appears to exhibit pleiotropic effects beyond psychiatric and autoimmune phenotypes. Previous studies also linked rs4702 to vascular traits, including systolic blood pressure [27, 28], which is consistent with our observation that it influences gene expression in arterial tissues (Supplementary Table 6).

Secondly, our functional assessments for candidate shared loci can be further refined. Limited availability of *trans*-eQTL and pQTL datasets constrained our ability to assess long-range regulatory effects and downstream molecular consequences, particularly in brain tissues. In this study, we did not detect significant SNP-protein associations in brain or cerebrospinal fluid (CSF). It remains unclear whether this reflects a true absence of effect or insufficient detection sensitivity. Future studies using high-sensitivity proteomic platforms, such as antibody-based Olink assays [29], and incorporating region-specific brain proteomics, will help resolve these uncertainties.

Also, due to restricted availability of individual-level GWAS data, we performed fine-mapping using summary statistics. For loci with multiple independent signals, approximate stepwise regression may introduce false positives and obscure the identification of true causal variants. Access to individual-level data in future research would improve mapping resolution.

Finally, while colocalisation and MR analyses provide insights into potential causal relationships, they do not establish causality. To validate the causal relationship between SCZ-SLE and protein and/or gene expression, especially in immune cells, extending the CRISPR-based allele conversion approach, previously applied in neuronal cells, to immune cell models could enable direct testing of allele-specific effects on gene/protein expression and measuring the functional consequences such as pro-inflammatory cytokine production.

### Therapeutic implications

The *FURIN-FES* locus presents limited opportunities for shared therapeutic targeting. Given its broad substrate specificity, FURIN itself is unlikely to represent a viable therapeutic target. Although FURIN substrate proteins (NTF4, GDF2, BMP10) did not show evidence for a causal role in our study, they remain candidates for future exploration, especially for GDF2 and BMP10 which may link to SLE through BMP signalling (Fig. 8). FES shows clearer involvement in immune regulation, though its role in neuronal processes remains uncertain. While its therapeutic potential is yet to be defined, the causal relationship between plasma FES levels and both diseases suggests potential utility as a shared biomarker.

In summary, we identify multiple loci contributing to the genetic overlap between SCZ and SLE, but show that these associations are mechanistically diverse and context-dependent. This underscores the need for cautious interpretation of cross-disease genetic analyses and prioritisation of functional validation to disentangle shared association from shared biology.

## Materials and methods

### Study design

This study utilized large scale genome-wide association study (GWAS) summary statistics to investigate the shared genetic architecture between SLE and SCZ. We estimated the overall genetic correlation between the two diseases using LD score regression and identified candidate shared loci using multiple methods. Loci that were candidates for harboring causal variants shared by both diseases were determined using colocalisation analysis, and their functional effects were evaluated using eQTL and pQTL data. To identify potential causal relationships, Mendelian randomization was employed. The overview of research design is shown in Supplementary Fig. 11.

### Study cohorts description

We utilized GWAS summary statistics from both published and unpublished studies, including datasets generated by our group and publicly available resources. The systemic lupus erythematosus (SLE) dataset included GWAS summary statistics from four GWAS and an Immunochip study [10], comprising over 10 000 patients and 30 000 healthy controls, making it one of the largest lupus studies conducted to date. The schizophrenia (SCZ) dataset included GWAS summary statistics from 35 476 patients and 46 839 healthy controls. Detailed cohort descriptions are provided below.

Systemic lupus erythematosus (SLE) GWAS dataset.

The SLE dataset included four GWAS studies and one Immunochip dataset, with all participants of European ancestry (Supplementary Table 9). Among these, the SLE-Main [7], SLE-Genentech [8], SLE-Slegen [9] and SLE Immunochip datasets [10] are from previously published studies. In addition, we included an unpublished European GWAS cohort, ‘SLE-GSA’.

The SLE-GSA dataset comprises 1251 SLE cases genotyped in-house on the Illumina Global Screening Array and 6830 controls from the English Longitudinal Study of Ageing (ELSA) genotyped on the Illumina HumanOmni 2.5 M array.

The four SLE GWAS were imputed using the Trans-Omics for Precision Medicine (TOPMed) imputation server. Prior to imputation, variants that did not meet any of these criteria were excluded; non-monoallelic variants, genotyping call rate ≥ 95%, minor allele frequency (MAF) ≥ 0.005, Hardy–Weinberg equilibrium p-values (HWE-P) > 1.0 × 10^−6^. The subjects who met any of the following criteria were excluded from the analyses: subjects with genotyping call rate < 0.95, subjects with gender mismatch between self-reported gender and genotype-based sex, those who were in a high degree of relatedness, showing PiHAT > 0.25 estimated by PLINK1.978. After pre imputation QC, genome alignment was checked using tools from the McCarthy group at http://www.well.ox.ac.uk/∼wrayner/tools/. Post imputation SNP were retained only if they passed Imputation quality score ≥ 0.7.

The summary association data for SLE was generated from our lab using individual level genotype data. Each study was analysed for association using a linear mixed model (RVTESTS). The linear mixed model accounted for population structure well: inflation factors (Lambda) were 1.001 (SLE-GSA data, Lambda_1000 = 1.000), 1.003 (SLE-Main data, Lambda_1000 = 1.001), 1.014 (SLE-Slegen data, Lambda_1000 = 1.016), 1.008 (SLE-Genentech data, Lambda_1000 =1.000).

### SLE GWAS meta-analysis

We performed the main analyses using summary statistics from two meta-analyses of the aforementioned SLE association data: One meta-analysis consisted of all four GWAS, including three published and one unpublished GWAS: SLE-Main, SLE-Genentech, SLE-Slegen and SLE-GSA cohorts (Referred as ‘SLE meta-analysis’ dataset in the main text); We implemented an inverse variance fixed effects model (METAL), with genomic control correction.

Another meta-analysis included the Immunochip study with the three published GWAS: SLE-Main, SLE-Genentech, SLE-Slegen and SLE-Immunochip. This meta-analysis datasets was kindly provided by our colleagues [30].

In all analyses, we only retained variants with INFO score ≥ 0.7, MAF ≥ 0.01.

A summary of the SLE datasets is provided in Supplementary Table 9.

### Schizophrenia (SCZ) GWAS dataset

The Schizophrenia (SCZ) GWAS summary association dataset was obtained from the Psychiatric Genomics Consortium (PGC) [6]. This dataset included 35 476 SCZ cases and 46 839 controls of European ancestry (Referred as ‘SCZ’ or ‘SCZ meta-analysis data’ in the main text). According to the original study, approximately 9.5 million post-imputation variants passed Quality Control criteria (INFO score ≥ 0.6, MAF ≥ 0.01, successfully imputed in ≥20 samples). We used the summary data of European ancestry for our analyses, only variants with INFO score ≥ 0.7, MAF ≥ 0.01 were retained for following analyses.

The SCZ dataset is summarized in Supplementary Table 9.

### Checking published associated loci across traits for shared associations

To create lists of published SCZ and SLE variants, we first gathered 524 published SCZ associated variants from the major SCZ GWAS studies listed on the Psychiatric Genomics Consortium (PGC) site [6, 25, 31–33], and 184 published SLE associated variants from major SLE GWAS studies [7, 34, 35].

We used the SCZ meta-analysis and SLE four-GWAS meta-analysis (SLE meta-analysis) as SLE summary association data. We checked all published SCZ variants’ association p-values in the SLE data and created a Quantile-Quantile (Q-Q) plot, and the P-value was adjusted using Benjamini-Hochberg (BH) method. SNPs passing the False discovery rate (FDR) threshold of 0.05 were taken as associated and annotated in the Q-Q plot, the mapped genes were provided by the original publication; the same approach was taken for SLE published SNPs’ associations in SCZ.

### Genetic correlation analysis

To assess the genome-wide genetic correlation (r_g_) between SCZ and SLE, we applied the cross-trait LD score regression (LDSC) software (version 1.0.1) [11, 36]. We used the SLE four GWAS meta-analysis (SLE meta-analysis) as the SLE dataset and the SCZ meta-analysis. We restricted to overlapping SNPs between the SLE and SCZ data. The extended MHC region (25–33 Mb) was excluded from the analysis. We used LD scores estimated from the 1000 Genome European samples supplied with the LDSC software; detailed preparation steps are described in the original publication of LDSC [11].

To estimate local genetic correlation, we used ρ-HESS software (version 0.5) [12], a method which estimates the local genetic correlation within genome partitions. To explore the genetic correlation at the MHC locus, we focused on the extended MHC region (25–33 Mb). To increase the power to detect local genetic correlations, we used the meta-analysis of SLE datasets that include SLE-Main, SLE-Genentech, SLE-Slegen and SLE-Immunochip. We used the genome partition file for LD-independent loci in the European population supplied with the software [37], and the reference panel from European 1000 genome populations [38].

### Cross-trait meta-analysis

We performed cross-trait meta-analysis between SLE and SCZ using the SLE four GWAS meta-analysis (SLE meta-analysis), as was used in the genome-wide genetic correlation analysis, and the SCZ meta-analysis dataset. The MHC region (25–33 Mb) and sex chromosomes were excluded from the analysis. Strand issues and allele matching were checked using Python code and the main analysis was conducted using METAL [39]. We performed two analyses: In one, the summary data of both diseases were analysed using a standard fixed-effect, inverse variance-based approach to meta-analysis which tests for candidate cross-trait signals with the same direction of allelic effect. In the second approach we flipped the association direction (1/OR) in the SCZ data and used a standard inverse variance-based approach to meta-analysis, which tests for candidate cross-trait signals with opposing directions of allelic effect. In both analyses we selected candidate shared associated SNPs with the following criteria: (1) SNPs reaching a significance threshold of P < 5 × 10^−08^ in meta-analysis AND (2) SNPs showing nominal association of P < 0.01 in both diseases AND (3) the direction of effect in both diseases was aligned after the appropriate effect coding.

### *Cis*/*trans*-eQTL data

We obtained cis-eQTL data on whole blood from the eQTLGen consortium (eQTLGen phase I) [14]. We extracted cis-eQTL data on isolated human immune cells from the eQTL catalogue [16] following the procedures described in a previous publication [40]. There were a total of 29 cis-eQTL datasets from the eQTL catalogue extracted, the cell types included Granulocyte, Monocytes, Macrophages, T cells, B cells and NK cells, detailed cell types are shown in Supplementary Table 5. At the tissue scale, we included cis-eQTL data from all available tissues at GTEx v8 [15] that were deposited on Google cloud (European individuals, data access time: May 2023). We also obtained cis-eQTL data for 8 major brain cell types derived from the prefrontal cortex, temporal cortex and white matter [17]. The cell types included oligodendrocyte precursor cells (OPCs), Inhibitory/Excitatory Neurons, Pericytes, Endothelial cells, Microglia, Astrocytes and oligodendrocyte.

### *Cis*-eQTL gene selection

To identify eGenes for testing, we performed eQTL mapping using FUMA [41], based on the summary statistics of SCZ and SLE data. For each colocalized candidate shared loci from the cross-trait meta-analysis, we first extracted the independent SCZ/SLE signal lead SNPs and used these as ‘pre-defined lead SNPs’ in FUMA. We also included SNPs from the 1000 Genome Phase 3 European populations that are in LD (r^2^ > 0.8) with the pre-defined lead SNPs. In the eQTL mapping, we checked all available eQTL datasets on FUMA except outdated GTEx V6/V7 data. The significant SNP-gene pairs (FDR < 0.05) were followed up in colocalisation analyses. We performed colocalisation analysis between all mapped eGenes at the candidate shared loci and each disease GWAS.

#### *trans*-eQTL gene selection

*trans*-eGenes were identified using the same approach as the identification of *cis*-eGenes. The SCZ/SLE GWAS signal lead SNPs, and SNPs in LD (r^2^ > 0.8) were used for identification of SNP-gene pairs. Significant SNP-gene pairs were followed up in colocalisation analyses.

### *Cis*/*trans*-pQTL data

We obtained Plasma pQTL data from the Pharma Proteomics Project of UK biobank (Referred as ‘UKB-PPP’ data in analysis) [20] and Plasma pQTL data from Icelanders of deCODE genetic (Referred as ‘decode-Iceland’ data in analysis) [19].

We also included pQTL data derived from the central nervous system, including Brain [21] and Cerebrospinal fluid (CSF) [22], which were obtained via communication with the corresponding authors of the respective studies.

#### *cis*-pQTL protein selection

*cis*-pQTL colocalisation analyses were performed at candidate shared loci that colocalized between SCZ and SLE. We focused on the pQTL data corresponding to the eGenes already implicated at each of these loci, using proteins encoded by these genes for colocalisation testing.

#### *trans*-pQTL protein selection

To identify *trans*-pQTL effects, we began with the lead SNPs from the SCZ and SLE independent signals. For each SNP, we identified its LD proxies using an r^2^ threshold of ≥0.6. These SNPs and proxies were then queried against each pQTL dataset, and only significant SNP-protein associations (FDR ≤ 0.05) were retained. The proteins from the identified SNP-protein pairs were then checked for colocalisation with the SCZ/SLE association signals. We also included proteins identified as *trans*-eGenes. Given the complex functional role of FURIN, we further extended our *trans*-pQTL analysis by including all known FURIN interactors, as identified from prior studies and curated in the STRING database [13]. A comprehensive list of these proteins is provided in Supplementary Table 10.

### Stepwise regression using COJO

To identify independently associated variants within loci of interest across the genome, we performed stepwise conditional analysis using GCTA-COJO (v1.94.1) [42] on SCZ meta-analysis, SLE four GWAS meta-analysis (SLE meta-analysis), eQTL and pQTL datasets. We applied the stepwise regression option (−-cojo-slct) to detect independent association signals within each locus. For each signal identified, we used the conditional analysis option (−-cojo-cond) to obtain its independent signals across a locus. Specifically, for a locus with *k* independent signals, we estimated the effect of signal *j* by conditioning on the remaining *(k − 1)* signals identified by --cojo-slct. We used the SLE-Main cohort as the LD reference panel. In loci where we identified evidence of more than one signal, we applied relaxed COJO conditioning (p < 1 × 10^−3^) to regress out potential sub-threshold independent signals, in order to satisfy COLOC’s single causal variant assumption and reduce the risk of model violation. These additional signals (p < 1 × 10^−3^) were not reported as associated in the study. This conditioning step was not applied in loci where only a single association signal was detected, such as at the *FURIN-FES* locus. We used the default collinearity cutoff at 0.9 and distance of 10 Mb. To reduce false-positives, we visually inspected the marginal association and conditional signals using LocusZoom plots. We only retained independent signals that remained distinct after conditional analysis and visual inspection of regional association plots.

### Colocalisation

For each candidate shared loci we obtained from the cross-trait meta-analysis, we performed colocalisation analysis on each independent signal estimated by COJO. We used COLOC version 4, which assumes one single causal variant. This analysis returns the posterior probability that two traits share the same causal variant. We used the SCZ meta-analysis and the SLE four GWAS meta-analysis (SLE meta-analysis) at each candidate shared loci.

Colocalisation was performed on disease/disease (SCZ/SLE) and Disease/QTL on all pairwise comparison of signals in each locus. For each colocalisation analysis performed, signals are identified as colocalized if the posterior probability of sharing the same causal variant (PP_H4_) > 0.5. We discuss in the paper association signals with more robust evidence of colocalisation (PP_H4_ > 0.8) and followed these up with functional interpretation. In each colocalisation analyses, COLOC also returns the posterior probability that a SNP is the shared causal variant (SNP.PP) where SNP.PP sums to one over all SNPs. For each signal we calculated credible sets of SNPs that contain the shared causal SNPs with probability P by including the minimal set of SNPs in the credible set whose SNP.PP values sum to over P (summing from the highest SNP.PP).

### Mendelian randomization (MR)

To assess the causal relationship between levels of proteins and disease, where the pQTL signal had evidence of coloc with SCZ/SLE signals, we performed two-sample bi-directional MR. We used SCZ meta-analysis and the SLE four GWAS meta-analysis (SLE meta-analysis) as input data for both diseases and the summary statistics of decode-iceland/UKB-PPP pQTL data as protein input data.

We firstly use proteins as exposures and SCZ/SLE as outcome (‘forward MR’). Then in reverse, we used SCZ/SLE as exposures and proteins as outcomes. (‘Reverse MR’).

Genetic instrument selection process.

For the instrumental variables (IV) selection process, we conducted multiple QC steps to ensure the selected IVs satisfied three core assumptions of MR: (1) The Relevance assumption—IV should strongly associate with exposure. (2) The independence assumption—No confounders between IV and exposure/outcome. (3) The exclusion restriction assumption—IV should only associate with outcome through exposure [43].

To satisfy the relevance assumption, we first selected candidate IVs as SNPs which associated with exposure(s) and at a genome-wide significant threshold (p < 5 × 10^−8^). Linkage disequilibrium (LD) clumping was then applied using PLINK (v1.90b6.24) [44], with window of 10 MB to ensure selected SNPs were valid IVs. Proxy SNPs were selected at LD threshold r^2^ > 0.8 to replace candidate IVs missing in the outcome data. The SLE-Main cohort was used in the LD calculation above. F-statistics were used at the end to confirm the strength of IVs, weak IVs (F-statistic < 10) were discarded.

To satisfy the independence assumption, we queried all remaining IVs that satisfied the relevance assumption, and SNPs in LD (r^2^ > 0.8) with them, using LDtrait [45] applied to potential confounders of both diseases, and manually exclude candidate IVs that associated with the confounders. For SLE, the confounders included periodontitis [46], plasma cathepsin B level [47], gut microbiome [48], selenium [49], circulating GDF-15 level [50] and high serum iron status [51]. For SCZ, the confounders included smoking [52], alcohol consumption [53], level of physical activity [54], body mass index (BMI) [55], and diabetes [56].

To satisfy the exclusion restriction assumption, we ran LDtrait and removed IVs that directly associated with Outcome.

For proteins exhibiting trans-effects (GDF2, BMP10, and NTF4), to avoid pleiotropic bias [57], we additionally applied a ‘cis-MR’ approach for instrumental variable (IV) selection, selecting only IVs located on the same chromosome as the genes encoding these trans-affected proteins.

However, the above methods are not sufficient to satisfy the MR assumptions (2) and (3), so in addition we ran a series of sensitivity analyses described below.

Sensitivity analysis.

For loci with multiple instrumental variables (IVs), we performed Mendelian randomisation (MR) analysis using the inverse-variance weighted (IVW) method. When only a single IV was available, the Wald ratio (WR) method was applied. To assess potential horizontal pleiotropy, we examined the intercept from the MR-Egger regression; a p-value > 0.05 was considered indicative of no evidence of directional pleiotropy [58]. We further used the Mendelian Randomization Pleiotropy RESidual Sum and Outlier (MR-PRESSO) method [59] to detect and remove horizontal pleiotropic outliers. To evaluate the influence of individual IVs on the causal estimates, leave-one-out analysis was conducted [60]. The Heterogeneity among IVs was assessed using Cochran’s Q test. Finally, to confirm the directionality of the inferred causal relationship, Steiger filtering was applied [61], and only results with the correct direction of effect were retained. The IV selection process was conducted using a combination of in-house shell and python scripts. The MR was conducted using ‘TwoSampleMR’ R Package version 0.6.4 [60, 61].

## Supplementary Material

Supplementary_Results_ddag099
