# Supplementary material for: Shared genetic architecture of schizophrenia and lupus identifies pleiotropic loci with distinct downstream mechanisms
Source: Hum Mol Genet. 2026 Oct 1;35(20):ddag099. doi: 10.1093/hmg/ddag099 (PMC13630456; doi:10.1093/hmg/ddag099)
Supplement: Supplementary file 1 [file supplementary_results_ddag099.docx]

**Shared genetic architecture of schizophrenia and lupus identifies pleiotropic loci with distinct downstream mechanisms.**

**Chen Gong^1^, Nick Dand****^1^, Amy Butler^1^, Ariella Amar^1^, Lu Liu^2^, Pinelopi Maria Triantafyllou^1^, David Morris^1,#,*^, Timothy Vyse^1,#^**

**^1^ Department of Medical & Molecular Genetics, King’s College London, St Thomas Street, London, United Kingdom, SE1 9RT**

**^2^ Department of Cardiovascular Surgery, First Affiliated Hospital of University of Science and Technology of China/Anhui Provincial Hospital, Lujiang Road, Hefei City, Anhui Province, China, 230001**

**^#^These authors contributed equally.**

***** [**david.l.morris@kcl.ac.uk**](mailto:david.l.morris@kcl.ac.uk)

**Supplementary Results**

***DOT1L* locus colocalisation with eQTL signals**

The shared association signal between SCZ and SLE at the *DOT1L* locus identified as a significant cis-eQTL for multiple genes across all the eQTL datasets analyzed. The eGenes identified at this locus include *PLEKHJ1*, *AMH*, *DOT1L*, *AP3D1*, and *SF3A2*. We further examined the colocalization of SCZ and SLE signals with these five eGenes within the *DOT1L* locus across the eQTL datasets in bulk tissues. Colocalization was observed with *PLEKHJ1* and *DOT1L* in the GTEx data but not in the other eQTL data.

*Tissue-specific eQTLs from GTEx V8*

For the SCZ-SLE shared signal at the *DOT1L* locus, we find evidence supporting the most likely causal SNP may also have effects on *DOT1L* and *PLEKHJ1* expression.

We found moderate evidence of colocalisation between the SCZ-SLE shared signals at the *DOT1L* locus and a *DOT1L* independent cis-eQTL signal in Transverse Colon (SCZ.coloc.PP_H4_ = 82.9%, SLE.coloc.PP_H4_ = 54.8%) (**Supplementary Figure 4**). The risk allele (A) of the most likely causal SNP rs141958336 for the SCZ-SLE shared association at *DOT1L* associated with decreased *DOT1L* expression.

We found the SCZ-SLE shared signal at *DOT1L* colocalized with *PLEKHJ1* *cis*-eQTL signals in multiple tissues from GTEx V8, including Esophagus Gastroesophageal Junction (SCZ.coloc.PP_H4_ = 78.4%, SLE.coloc.PP_H4_ = 87.6%) (**Supplementary Figure 5-a**), Aorta artery (SCZ.coloc.PP_H4_ = 93.4%, SLE.coloc.PP_H4_ = 83.1%) (**Supplementary Figure 5-b**), Mammary tissue of breast (SCZ.coloc.PP_H4_ = 90.6%, SLE.coloc.PP_H4_ = 81%) (**Supplementary Figure 5-h**), cultured fibroblast (SCZ.coloc.PP_H4_ = 88%, SLE.coloc.PP_H4_ = 90.5%) (**Supplementary Figure 5-i**), sigmoid colon (SCZ.coloc.PP_H4_ = 86.5%, SLE.coloc.PP_H4_ = 87%) (**Supplementary Figure 5-e**), Adipose Visceral Omentum (SCZ.coloc.PP_H4_ = 76.6%, SLE.coloc.PP_H4_ = 83.8%) (**Supplementary Figure 5-d**), Tibial artery (SCZ.coloc.PP_H4_ = 51.8%, SLE.coloc.PP_H4_ = 92.8%) (**Supplementary Figure 5-c**), Brain Cerebellum (SCZ.coloc.PP_H4_ = 82.6%, SLE.coloc.PP_H4_ = 81.7%) (**Supplementary Figure 5-f**) and Adipose Subcutaneous (SCZ.coloc.PP_H4_ = 69.3%, SLE.coloc.PP_H4_ = 80.3%) (**Supplementary Figure 5-g**).

For all colocalized cis-eQTLs tissues above, the risk allele (A) of the most likely causal SNP rs141958336 for SCZ-SLE shared association *DOT1L* associated with decreased *PLEKHJ1* expression.

**Shared association at *FURIN-FES locus and its functional consequences*** **on *FURIN*, extended results.**

The SCZ-SLE shared signal at *FURIN-FES* shows complex regulatory effects on FURIN transcript level. At the tissue-level, the rs4702 disease risk allele (G) associated with weak increased *FURIN* expression in ascending aorta. However, at single-cell level, the same disease risk allele associated with decreased *FURIN* expression in blood isolated naïve monocytes, neutrophils, and brain excitatory neurons. The differences in SCZ-SLE shared signal effects on *FURIN* expression at tissue and single-cell level suggest cell-type-specific regulatory effects.

It is noteworthy that rs4702 is in the 3'UTR of *FURIN*, raising the possibility of post-transcriptional regulation. We found no evidence of histone modification across 21 cell lines or transcription factor binding modulation, based on data from ENCODE project [1] (**Supplementary Figure 7**), but there was evidence from GTEx data suggests rs4702 might be a sQTL for *FURIN* in tibial artery and leg skin exposed to the sun (**Supplementary Figure 8**). One possible regulatory mechanism of rs4702 covered in prior research suggested the G allele of rs4702 may create a binding site for a microRNA: miR-338-3p, which leads to reduced *FURIN* expression in Human embryonic kidney cell line (HEK293T) [2]. A CRISPR-based A to G allele conversion of rs4702 in neurons has further shown that switching from homozygous A to homozygous G at rs4702 reduced *FURIN* expression in excitatory neurons [3], which was also confirmed in our study. Interestingly, in neural progenitor cells (NPCs), the homozygous rs4702 G allele increased *FURIN* expression, while no significant effects were observed in other neuronal subtypes. With respect to phenotype changes related to the rs4702 A to G allele conversion, it was shown that homozygous G allele compared with homozygous A allele in excitatory neurons, also associated with significantly decreased neurite length and neuron activity [3].

At the proteomics level, *FURIN* encodes a proprotein convertase, and plays a role in cleaving pro-proteins into active form, including TGF-β superfamily members. We showed that there was colocalisation between SCZ / SLE association signals and pQTL signals of FURIN and some of its known targets, including TGF-β superfamily members: GDF2, BMP10, and a Neurotrophic factor NTF4. All three proteins are known to interact with FURIN or contain FURIN cleavage sites [4-6]. The disease risk allele (G) of rs4702 correlated with decreased FURIN, GDF2, BMP10 and NTF4 levels in plasma.

The MR results did not support a causal relationship between FURIN protein levels in serum and both diseases. This was not surprising because, although FURIN has broad roles in embryogenesis, homeostasis and diseases such as psychiatric disorders, cancer and infection, its role in these events largely stems from the generation of the biologically active form of the substrates [7].

We are unable to assess causal relationships between transcript levels (eQTLs) and diseases, this is due to the absence of valid IVs in both *cis*- and *trans*-eQTL data. We also conservatively focus on cis-acting pQTLs (rather than *trans*-pQTL) because of their greater reliability indicated in part by use in phenome-wide scans to investigate drug targets [8, 9]. In contrast, *trans*-acting pQTLs often influence protein levels through indirect or complex mechanisms and are therefore more likely to be pleiotropic, hence risk violating MR assumptions [10]. Recent work has suggested that the pleiotropic risk of *trans*-pQTLs could be mitigated by restricting trans-acting instrumental variables to those associated with limited proteins across pQTL datasets [11]. Although such stringent filtering may reduce the likelihood of pleiotropic bias, it can also inflate the risk of false-negative findings by excluding biologically relevant but moderately pleiotropic instruments, and it is difficult to define a non-arbitrary ‘cutoff’ for the number of proteins the IVs are associated with for specific proteomics data. Future studies with more comprehensive proteome-wide datasets and advanced strategies for *trans*-pQTL evaluation may enable a better assessment of trans-mediated causal pathways. An exploratory ‘cis-MR’ analyses, restricting instruments to SNPs on the same chromosome as the *trans*-acting proteins (GDF2, BMP10, NTF4) did not reveal causal effects in either direction. These null findings suggest that, within the constraints of current pQTL resources, there is no support for a direct causal role of BMP10, GDF2, or NTF4 with protein levels in SCZ or SLE.

With reference to biological mechanisms of the shared signal at *FURIN-FES* in both diseases, reduced *FURIN* expression in excitatory neurons has been linked to impaired neuronal growth and activity [3], disrupting the balance between excitatory and inhibitory neuronal activity in the medial temporal lobe and has been implicated in SCZ pathogenesis [12]. Although neurotrophin-4 (NT-4) and other neurotrophins are known to promote neuronal survival and synaptic plasticity in the central nervous system [4], there is currently no evidence that plasma NT-4 exerts similar biological effects. Decreased plasma NT-4 level therefore likely reflects the impact of disease state. In immune cells, the association between the shared signal at *FURIN-FES* and reduced plasma levels of two downstream FURIN substrates, GDF2 and BMP10, suggests an intriguing indirect connection to immune regulation. Both GDF2 and BMP10 are members of the TGF-β superfamily and are components of BMP signaling, which mostly associate with development and tissue homeostasis [13]. But notably, recent evidence implicates GDF2 in rheumatoid arthritis (RA), a condition closely related to SLE, where its downregulation in synovial tissue correlates with enhanced PI3K/AKT signaling in fibroblast-like synoviocytes (FLSs) and promotes cell proliferation and migration [14]. These observations support an anti-inflammatory role for GDF2 and implicate BMP signaling in the FURIN-SLE association via immune cells. In summary, the effect of the SCZ-SLE shared signal at the *FURIN-FES* locus on FURIN suggests an association with both diseases via distinct pathways in different cell types: A SCZ-associated neuronal pathway in neuronal cells and SLE-associated immune regulatory pathway in immune cells (**Figure 8**).

**Shared association at *FURIN-FES locus and its functional consequences*** **on *FES*, extended results.**

The SCZ and SLE association signals both colocalised with multiple *FES* eQTL in immune-related cells. In all cell types noted, the disease risk allele associated with increased *FES* expression. Compared with the *FURIN* cis-eQTL, we observed association peaks in the *FES* cis-eQTL in many more immune cell types within the narrow region (± 200 Kb) surrounding the SCZ/SLE independent signals (**Supplementary Figure 9,** see ‘Likely shared’ in the evidence of sharing column). Notably, no *FES* eQTL association peaks were observed in any brain tissues or neuronal cells.

Unlike *FURIN*, the SCZ-SLE shared association tagging SNP rs4702 is a significant sQTL for *FES* in over 20 human tissues, suggesting its potential involvement in alternative splicing (**Supplementary Figure 10**), but the exact splice event requires further investigation. Additionally, rs4702 is located in the *FES* promoter (*FES* is adjacent to *FURIN* and coded on the same strand), raising the possibility that it modulates *FES* transcription (**Supplementary Figure 7**). The exact regulatory effects of rs4702 on *FES* expression remain poorly understood.

At the protein level, we observed colocalisation between the SCZ/SLE shared association and a *cis*-pQTL signal of FES, and a *trans*-pQTL signal for the functional analogue, FER. The rs4702 risk allele associated with increased FES and FER protein levels in the plasma (**Supplementary Table 7**). The MR analysis revealed a positive causal relationship between FES protein level and both SCZ / SLE. We did not find evidence for causal relationship between the FER protein level and SCZ/SLE in either direction using the experimental ‘cis-MR’ method.

The shared signal at *FURIN-FES* appears to influence *FES* transcript levels specifically in immune cells, with no evidence of regulatory effects in neuronal tissues or cells (**Supplementary Figure 9**). Alterations in *FES* expression have been linked to immune modulation, as previous research has shown that *FES* knockout mice displayed defective TLR4 internalization and associated with hypersensitivity upon LPS challenge [15]. *FES* has also been correlated with suppressed TLR-related inflammatory responses in the MHC class-I axis [16], and may act as a novel intracellular innate immune checkpoint [17]. Interestingly, the upregulation of *FES* observed in our results contrasts with its previously proposed anti-inflammatory role [18]. The FER protein also plays a role in supressing immune response but through different mechanism. The FER protein, a tyrosine kinase closely related to FES, shares similar biological functions but acts through a distinct mechanism. FER has been shown to limit inflammation by promoting STAT3 activation and enhancing IL-10 signalling in macrophages, thereby suppressing proliferation and reducing pro-inflammatory cytokine production [18].

Our evidence for a positive causal relationship between plasma FES levels and SLE risk, suggest that FES may contribute to unknown pro-inflammatory mechanisms involved in SLE pathogenesis. Alternatively, increased *FES* expression may represent a compensatory response to heightened immune activation (**Figure 8**). While MR analysis also suggested a positive causal association between FES levels and SCZ risk, we found no eQTL evidence for *FES* expression in brain tissues or neuronal cells (**Supplementary Figure 9**), and there is no prior evidence implicating FES in neuronal function. Therefore, whether FES truly plays a causal role in SCZ pathogenesis remains an open question and requires further validation. The above results suggest the effect of the SCZ-SLE shared signal at *FURIN-FES* locus on *FES* likely exerts a role in SLE pathogenesis via immune cells.

**References**

1.	Dunham, I., A. Kundaje, S.F. Aldred, P.J. Collins, C.A. Davis, F. Doyle, C.B. Epstein, S. Frietze, J. Harrow, R. Kaul, et al., *An integrated encyclopedia of DNA elements in the human genome.* Nature, 2012. **489**(7414): p. 57–74.

2.	Hou, Y., W. Liang, J. Zhang, Q. Li, H. Ou, Z. Wang, S. Li, X. Huang, and C. Zhao, *Schizophrenia-associated rs4702 G allele-specific downregulation of FURIN expression by miR-338-3p reduces BDNF production.* Schizophrenia Research, 2018. **199**: p. 176–180.

3.	Schrode, N., S.-M. Ho, K. Yamamuro, A. Dobbyn, L. Huckins, M.R. Matos, E. Cheng, P.J.M. Deans, E. Flaherty, N. Barretto, et al., *Synergistic effects of common schizophrenia risk variants.* Nature Genetics, 2019. **51**(10): p. 1475–1485.

4.	Zhang, Y., X. Gao, X. Bai, S. Yao, Y.-Z. Chang, and G. Gao, *The emerging role of furin in neurodegenerative and neuropsychiatric diseases.* Translational Neurodegeneration, 2022. **11**(1): p. 39.

5.	Susan-Resiga, D., R. Essalmani, J. Hamelin, M.-C. Asselin, S. Benjannet, A. Chamberland, R. Day, D. Szumska, D. Constam, S. Bhattacharya, et al., *Furin Is the Major Processing Enzyme of the Cardiac-specific Growth Factor Bone Morphogenetic Protein 10.* Journal of Biological Chemistry, 2011. **286**(26): p. 22785–22794.

6.	Hernandez, F., R. Huether, L. Carter, T. Johnston, J. Thompson, J.R. Gossage, E. Chao, and A.M. Elliott, *Mutations in RASA1 and GDF2 identified in patients with clinical features of hereditary hemorrhagic telangiectasia.* Human Genome Variation, 2015. **2**(1): p. 15040.

7.	Thomas, G., *Furin at the cutting edge: From protein traffic to embryogenesis and disease.* Nature Reviews Molecular Cell Biology, 2002. **3**(10): p. 753–766.

8.	Millwood, I.Y., D.A. Bennett, M.V. Holmes, R. Boxall, Y. Guo, Z. Bian, L. Yang, S. Sansome, Y. Chen, H. Du, et al., *Association of CETP Gene Variants With Risk for Vascular and Nonvascular Diseases Among Chinese Adults.* JAMA Cardiology, 2018. **3**(1): p. 34–43.

9.	*The interleukin-6 receptor as a target for prevention of coronary heart disease: a mendelian randomisation analysis.* The Lancet, 2012. **379**(9822): p. 1214–1224.

10.	Zheng, J., V. Haberland, D. Baird, V. Walker, P.C. Haycock, M.R. Hurle, A. Gutteridge, P. Erola, Y. Liu, S. Luo, et al., *Phenome-wide Mendelian randomization mapping the influence of the plasma proteome on complex diseases.* Nature Genetics, 2020. **52**(10): p. 1122–1131.

11.	Yang, C., A.M. Fagan, R.J. Perrin, H. Rhinn, O. Harari, and C. Cruchaga, *Mendelian randomization and genetic colocalization infer the effects of the multi-tissue proteome on 211 complex disease-related phenotypes.* Genome Medicine, 2022. **14**(1): p. 140.

12.	Harrison, P.J. and S.L. Eastwood, *Preferential involvement of excitatory neurons in medial temporal lobe in schizophrenia.* The Lancet, 1998. **352**(9141): p. 1669–1673.

13.	Akiyama, T., L.A. Raftery, and K.A. Wharton, *Bone morphogenetic protein signaling: the pathway and its regulation.* Genetics, 2023. **226**(2).

14.	Song, B., X.-F. Li, Y. Yao, Q.-Q. Xu, X.-M. Meng, C. Huang, and J. Li, *BMP9 inhibits the proliferation and migration of fibroblast-like synoviocytes in rheumatoid arthritis via the PI3K/AKT signaling pathway.* International Immunopharmacology, 2019. **74**: p. 105685.

15.	Parsons, S.A. and P.A. Greer, *The Fps/Fes kinase regulates the inflammatory response to endotoxin through down-regulation of TLR4, NF-κB activation, and TNF-α secretion in macrophages.* Journal of Leukocyte Biology, 2006. **80**(6): p. 1522–1528.

16.	Xu, S., X. Liu, Y. Bao, X. Zhu, C. Han, P. Zhang, X. Zhang, W. Li, and X. Cao, *Constitutive MHC class I molecules negatively regulate TLR-triggered inflammatory responses via the Fps–SHP-2 pathway.* Nature Immunology, 2012. **13**(6): p. 551–559.

17.	Laight, B.J., D. Harper, N. Dmytryk, S. Zhang, A. Garven, C. Shi, R. Nauman, J. Kment, F. Alotaibi, I. Shapavalov, et al., *Fes-deficient macrophages enhance CD8<sup>+</sup> T cell priming and tumour control through increased pro-inflammatory cytokine production and localization.* bioRxiv, 2024: p. 2024.02.27.581601.

18.	Greer, P., *Closing in on the biological functions of fps/fes and fer.* Nature Reviews Molecular Cell Biology, 2002. **3**(4): p. 278–289.
